# Association of Pneumatosis Intestinalis With Surgical Outcomes and Mortality: A Matched, Retrospective Cohort Study and Literature Review

**DOI:** 10.1097/AS9.0000000000000448

**Published:** 2024-06-21

**Authors:** Kyle D. Klingbeil, Hila Zelicha, Yijun Chen, Douglas S. Bell, Edward H. Livingston

**Affiliations:** * From the Department of Surgery, UCLA David Geffen School of Medicine, Los Angeles, CA; † Department of Medicine, Division of General Internal Medicine, UCLA, Los Angeles, CA; ‡ Informatics Program of the UCLA Clinical and Translational Science Institute (CTSI), UCLA, Los Angeles, CA.

**Keywords:** acute care surgery, general surgery, outcomes research, pneumatosis intestinalis

## Abstract

**Background:**

To determine the clinical importance of pneumatosis intestinalis (PI) on surgical decision-making and patient outcomes.

**Methods:**

A matched cohort observational study was conducted including all clinical encounters for both ambulatory and inpatient care at UCLA Health between February 15, 2006 and January 31, 2023. Patients were initially identified using encounter diagnostic codes for “other specified diseases of intestine.” A radiologic diagnosis of PI was then assessed using natural language processing techniques followed by confirmation using manual chart review. Patients who did not have PI served as a control group. Patient comorbidity was assessed using Elixhauser comorbidity scores. Logistic regression and Cox hazard analyses were used to assess associations between PI and mortality. The main outcome was 90-day all-cause mortality. Secondary outcomes were the proportion of patients undergoing surgery and, of those, how many required bowel resections.

**Results:**

Of the 16,728 patients identified by diagnostic coding, 315 were confirmed to have a diagnosis of PI. The 90-day mortality rate for all patients with PI was 29%. Surgery was performed for 62 patients (20%), of whom 46 (72%) underwent bowel resection and 16 (28%) underwent abdominal exploration alone. Most patients underwent surgery for peritonitis (37%), bowel obstruction (31%), and/or pneumoperitoneum (23%) in association with PI; whereas only 8% of patients received surgery exclusively for PI. There was no statistically significant association between PI and mortality with logistic regression conditioned on other risk factors for mortality. In contrast, survival analysis of a matched cohort demonstrated a small effect of PI on mortality (hazard ratio = 1.24: 95% confidence interval = 1.16–1.32, *P* = 0.021).

**Conclusions:**

Most patients with a diagnosis of PI survive without requiring surgery. Of those who undergo surgery, nearly all have indications for laparotomy exclusive of PI. Mortality in patients who have pneumatosis is strongly associated with comorbid disease, with little to no independent association with PI. Our findings suggest that the presence of PI should not be a primary indication for surgical intervention.

## INTRODUCTION

The presence of gas within the wall of the intestinal tract, also known as pneumatosis intestinalis (PI), is an increasingly common radiologic finding of uncertain significance. PI is usually a self-limiting condition, but in some cases, is associated with more serious pathology such as bowel necrosis and/or sepsis.^1,2^ As a result, the optimal management of PI is unknown.

Because of the association between PI and life-threatening conditions, PI itself may be considered as an independent indication for surgical exploration of the abdomen.^2–10^ However, because most cases of PI are self-limiting, surgical intervention is rarely warranted. Having tools predicting the need for surgery when PI is present might reduce the time from diagnosis to surgery and prevent morbidity from unnecessary surgical interventions. Consequently, most prior studies examining outcomes for PI have sought factors predictive of ischemic bowel to help guide decision-making regarding whether to pursue surgical exploration of the abdomen.^11–23^ Most of these studies showed that nonspecific clinical findings such as peritonitis or lactic acidosis were indicators warranting surgery. Given these studies failed to identify factors specific to PI, we hypothesized that PI itself does not predict the need for bowel resection or patient death.

To test this hypothesis, we examined outcomes for all patients who received a radiologic diagnosis of PI and reviewed their outcomes at our institution during the past 17 years. A matched cohort was constructed to determine the effect of PI on 90-day overall survival (OS). Operative notes and pathology reports were reviewed for patients who underwent surgery to assess indications for surgery and how those reconciled with operative and pathological findings.

## METHODS

### Cohort Selection and Natural Language Processing

The records of all patients receiving healthcare at UCLA Health between February 15, 2006 and January 31, 2023 were available for analysis. Age, sex, race, and ethnicity were extracted from the UCLA Epic Clarity electronic health record database that includes all encounters at our institution, after the removal of restricted patients such as celebrities. No International Classification of Diseases (ICD) 9th/10th revision code exists for PI. Therefore, our initial search included records coded as “other specified disorders of the intestines” (ICD-9 569.89 or ICD-10 K63.89).

PI cases were identified from this initial cohort using natural language processing techniques applied to the text narratives of radiographic imaging reports. The reports were broken down into individual sentences and converted to lowercase letters. Word searches were conducted using R module “grepl” for sentences containing the word “pneumatosis” and the misspelled word “pneumotosis.” Whether the word pneumatosis was associated with a positive finding (eg, “pneumatosis was present”) or negative (eg, “no pneumatosis was seen”) was determined using R package “lexicon” with the module “hash_valence_shifters” that identified word negators, amplifiers, deamplifiers, and adversative conjunctions. See Supplemental text, see http://links.lww.com/AOSO/A355 for R code used in this study. The automated classification was followed by a manual review of the positive/negative classification by 2 authors (K.D.K. and E.H.L.) to ensure accurate classification. In cases when pneumatosis was observed in more than one image spanning more than 1 day, the date of the image when pneumatosis was first identified was established as the date of diagnosis.

Procedure files were then matched to patients with a diagnosis of PI. Procedures were classified as having an operation associated with bowel resection, laparotomy without bowel resection, or no surgery. The time, in days, between the first diagnosis of PI and the day of surgery was recorded. Pathology files were reviewed to confirm operative findings as stated in the operative notes. The operative note indications were reviewed and classified as PI only, pneumoperitoneum, peritonitis, bowel obstruction, hemodynamic instability, or bleeding. If patients died while hospitalized, mortality data were available and the date between the first diagnosis of PI and patient death was recorded. Mortality was assessed as a continuous variable for some statistical analyses and at 30 or 90 days from the first diagnosis of PI. Laboratory values such as white blood cell (WBC), bicarbonate, and lactate were also examined. Laboratory values were entered into the analysis if they were obtained on the day of or up to 2 days prior to the first PI diagnosis. When there were multiple laboratory values for this time interval, the maximum (eg, WBC, lactate) or minimum (eg, bicarbonate) values were selected, depending on which direction reflected the most pathologic state.

### Cohort Matching

All diagnoses coded for billing within all encounters were available for review. From these, diagnoses associated with being present on admission or the primary cause for hospital admission were used for comorbidity analysis. Elixhauser comorbidity scores were calculated using van Walraven weights with the R package comorbidity (https://cran.r-project.org/web/packages/comorbidity/comorbidity.pdf).^24^ The comorbidity “fluid and electrolyte disorders” was reported but not included in statistical calculations because this can be a consequence of ischemic bowel and a cause for hospital admission.

A matched cohort was generated by matching PI patients to non-PI patients who were in the “other specified disorders of intestines” cohort using the R module MatchIt. Match methods included nearest neighbor, optimal, full, coarsened exact, and exact matching. The best match was selected as the one that had the fewest unmatched PI patients and the shortest generalized weighted distance between the 2 cohorts (https://cran.r-project.org/web/packages/MatchIt/vignettes/assessing-balance.html). Balance was sought between the groups for Elixhauser comorbidities, patient age at the time of diagnosis, and sex assigned at birth. An absolute number representing the number of days between the date of first diagnosis of PI and January 1, 1970 was used to match the date of a PI diagnosis and when a computed tomography (CT) was performed in the control group.

### Statistical Techniques

The association between the presence of pneumatosis and 90-day mortality was assessed by Cox regression of the matched cohort with the presence or absence of pneumatosis as the independent variable. Logistic regression of 90-day mortality on pneumatosis, age, male sex, and the Elixhauser comorbidity variables was performed to determine the association of these conditions on mortality. The magnitude of differences between groups was assessed by calculation of standardized mean differences (SMD). All data extraction and statistical analyses were performed using R Studio Version 2023.09.1 Build 494 and R version 4.3.1.

### Literature Review

A literature search in PubMed was conducted for articles published between 2018 and 2023 using the search term “pneumatosis.” Articles reporting 40 or more patients with PI were considered for in-depth review. Reference lists from available articles were also reviewed to query articles not captured in the initial PubMed search.

## RESULTS

**FIGURE 1.**
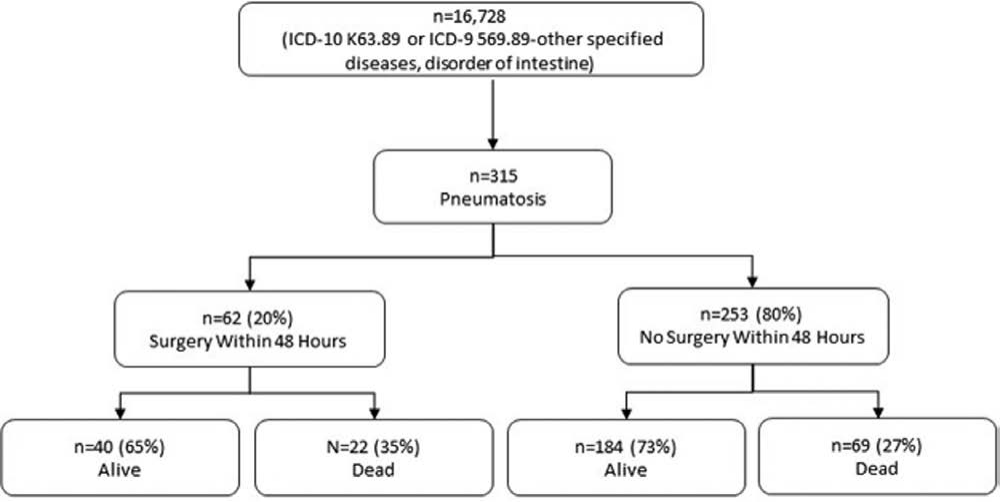
Flow diagram of patients screened and included in the study along with the proportion of those undergoing surgery and their survival.

Between February 15, 2006 and January 31, 2023, there were 16,728 patients who were coded as having ICD-10 K63.89 or ICD-9 569.89 (other specified diseases/disorder of intestine) at our institution. These patients underwent 425,832 radiographic examinations during the study period, which resulted in 7,431,222 individual lines of text from imaging reports to be processed. Using natural language processing methods followed by manual review, we identified 315 patients with 621 imaging reports demonstrating findings of PI (Fig. 1). There were 621 patients who had 696 radiology reports that specifically mentioned PI was absent.

Baseline characteristics of the cohort with PI are summarized in Table 1. Of the 315 patients with PI, 91 (29%) died within 90 days of a diagnosis. Patients who died tended to be older, male, identified as White race, Hispanic ethnicity, and had elevations in WBC or lactate with lower levels of bicarbonate.

**TABLE 1.** Baseline Characteristics of Patients Who Experienced Pneumatosis Intestinalis

	Survive (n = 224)	Dead (n = 91)	SMD*
Age, y			
Mean (SD)	64.2 (17.6)	57.1 (17.0)	0.41
Sex			
Male	83 (35.5%)	54 (65.1%)	0.62
Race			
White or Caucasian	80 (34.2%)	47 (56.6%)	0.463
Asian	14 (6.0%)	6 (7.2%)	0.05
Black or African American	18 (7.7%)	9 (10.8%)	0.109
Middle Eastern or North African	0 (0%)	1 (1.2%)	0.156
Other	50 (21.4%)	20 (24.1%)	0.065
Ethnicity			
Hispanic, n (%)	45 (19.2%)	29 (34.9%)	0.94
Any laparotomy, n (%)	39 (16.7%)	18 (21.7%)	0.13
Exploratory laparotomy			
No bowel resection, n (%)	12 (5.1%)	5 (6.0%)	0.039
Any bowel resection, n (%)	27 (11.5%)	13 (15.7%)	0.12
WBC, cells/µL			
Mean (SD)	10.3 (7.5)	15.5 (11.8)	0.53
Lactate, mg/dL			
Mean (SD)	20.8 (17.2)	47.9 (34.4)	1
CK, U/L			
Mean (SD)	666.1 (1915.1)	879.5 (2134.3)	0.1
Troponin			
Positive, n (%)	7 (3.0%)	10 (12.0%)	0.255
Base excess, mmol/L			
Mean (SD)	−2.2 (5.2)	−2.9 (5.2)	0.14
Bicarbonate, mmol/L			
Mean (SD)	21.4 (5.6)	18.1 (5.6)	0.58

Laboratory values are those collected on the day or 2 days before the first radiologic diagnosis of pneumatosis. If there were multiple values, the highest or lowest (base excess and bicarbonate) values were assigned to the patient. Death represents those patients dying at a UCLA facility within 90 days of the first pneumatosis diagnosis.

* Small effect SMD <0.5; medium effect, 0.5–0.8; large effect SMD >0.8.

CK indicates creatine kinase.

Of the 315 patients with pneumatosis, 74 (23%) underwent surgery and 241 (77%) did not (Table 2). Of those who received an operation, 51 (69%) underwent a bowel resection, whereas 23 (31%) underwent a negative laparotomy, and 62 (84%) had their operations within 48 hours of PI diagnosis. The remaining 12 (16%) with delayed surgery had operations for unrelated problems such as bowel obstruction without ischemia (n = 6) or had a delayed presentation of ischemic bowel masked by other problems such as portal hypertension or pneumonia.

**TABLE 2.** Survival and Surgery Outcomes for Patients With Pneumatosis Intestinalis

	Alive (n = 224)	30-D Mortality (n = 71)	90-D Mortality (n = 91)
No surgery (n = 241)	175 (78%)	54 (76%)	66 (73%)
Surgery ≤48 h (n = 62)			
Exploratory laparotomy			
No bowel resection (n = 16)	13 (6%)	3 (4%)	3 (3%)
Bowel resection (n = 46)	27 (12%)	13 (18%)	19 (21%)
Surgery>48 h (n = 12)			
Exploratory laparotomy			
No bowel resection (n = 7)	5 (2%)	2 (3%)	2 (2%)
Bowel resection (n = 5)	4 (2%)	0 (0%)	1 (1%)

Percentages are relative to the survival status.

The major indications for surgery are summarized in Table 3. Hemodynamic instability (55%), peritonitis (37%), bowel obstruction (31%), pneumoperitoneum (23%), or bleeding (3%) were major indications for surgery. Only 5 (8%) patients underwent laparotomy exclusively for a diagnosis of pneumatosis. Of these patients, 2 required bowel resection and 3 received a negative laparotomy.

**TABLE 3.** Indications for Surgery for Patients Undergoing Surgery Within 48 Hours of Initial Findings of Pneumatosis Intestinalis

Indication	Number (%) (n = 62)*
Hemodynamic instability	34 (55)
Peritonitis	23 (37)
Bowel obstruction	19 (31)
Pneumoperitoneum	14 (23)
Pneumatosis alone	5 (8)
Bleeding	2 (3)

* Patients may have had multiple indications for surgery such that the percentages do not total 100%.

Risk factors for mortality were assessed by the Elixhauser comorbidity system and scored by the van Walraven method (Supplemental Table 1, see http://links.lww.com/AOSO/A355).^24^ Because of missing data, this analysis was performed on the 277 (88%) patients who had complete encounter ICD coding information available. Of these 277, 92 (33%) died in-hospital within 90 days of PI diagnosis. The analysis was restricted to diagnostic codes labeled as present on admission or the primary diagnosis for the index admission. There was a strong association between the van Walraven-Elixhauser score and 90-day mortality (SMD = 0.96). Of the individual components of the Elixhauser score, fluid and electrolyte disorders and alcohol abuse had the strongest association with mortality with moderately large SMDs (SMD = 0.52, 0.52, respectively).

Logistic regression was used to determine the association between the various risk factors and 90-day mortality (Table 4 and Supplemental Table 2, see http://links.lww.com/AOSO/A355). The strongest associations were between metastatic cancer (odds ratio, 4.78; 95% confidence interval [CI], 4.23–5.40), lymphoma (2.39; 1.95–2.94), liver disease (2.26; 2.10–2.43), complicated hypertension (1.91; 1.73–2.10), and other neurologic disease (1.89; 1.75–2.05) and 90-day mortality. Following adjustment by the various comorbid conditions, PI was not significantly associated with mortality.

**TABLE 4.** Logistic Regression of Risk Factors for 90-Day Mortality in Patients With a Diagnosis of Pneumatosis

Characteristic	Odds Ratio	95% CI	*P* Value
Metastatic cancer	4.78	4.23–5.40	<0.001
Lymphoma	2.39	1.95–2.94	<0.001
Liver disease	2.26	2.10–2.43	<0.001
Complicated HTN	1.91	1.73–2.10	<0.001
Other neurologic disease	1.89	1.75–2.05	<0.001
Paralysis	1.71	1.46–2.00	<0.001
Coagulopathy	1.57	1.46–1.70	<0.001
Peripheral vascular disease	1.52	1.41–1.64	<0.001
Solid tumor	1.46	1.34–1.60	<0.001
Rheumatoid arthritis/CVD	1.45	1.30–1.62	<0.001
Weight loss	1.39	1.29–1.50	<0.001
Renal failure	1.38	1.27–1.51	<0.001
Blood loss anemia	1.37	1.12–1.67	0.002
Uncomplicated diabetes	1.36	1.23–1.50	<0.001
Drug abuse	1.32	1.16–1.52	<0.001
Complicated diabetes	1.2	1.09–1.32	<0.001
Sex (male)	1.12	1.05–1.20	<0.001
CHF	1.12	1.03–1.23	0.011
Time first pneumatosis Dx	1	1.00–1.00	<0.001
Obesity	0.89	0.79–1.01	0.071
Uncomplicated HTN	0.87	0.81–0.94	<0.001
Pulmonary circulation disorders	0.84	0.76–0.94	0.002
Chronic pulmonary disease	0.71	0.65–0.77	<0.001
AIDS	0.67	0.46–0.97	0.035
Alcohol abuse	0.66	0.59–0.73	<0.001
Peptic ulcer disease	0.5	0.42–0.61	<0.001
Psychoses	0.17	0.13–0.21	<0.001
Cardiac arrhythmias	1.03	0.96–1.11	0.4
Depression	1.01	0.93–1.11	0.7
Age	1	1.00–1.00	0.4
Deficiency anemia	0.98	0.89–1.08	0.7
Valvular disease	0.97	0.88–1.08	0.6
Hypothyroidism	0.97	0.88–1.07	0.5
Pneumatosis intestinalis	0.3	0.01–18.6	0.6

CVD indicates collagen vascular disease; HTN, hypertension.

A matched cohort was created from the 16,728 patients who had diagnostic codes for “other specified disorders of intestines” and confirmed the presence or absence of PI on radiographic imaging reports. The treatment group of the cohort were those patients who had a radiologic diagnosis of PI with all other patients being considered controls. There was sufficient information in our database to match 287 of the 315 pneumatosis patients. No match was found for the exact matching method and only 26 patients were matched using coarsened exact matching. All cases were matched with nearest neighbor, optimal, and full matching with the shortest distance between groups being with full matching. The full matched cohort resulted in an excellent match (Supplemental Figure 1, see http://links.lww.com/AOSO/A355 and Supplemental Table 3, see http://links.lww.com/AOSO/A355) and was used for subsequent analyses.

**FIGURE 2.**
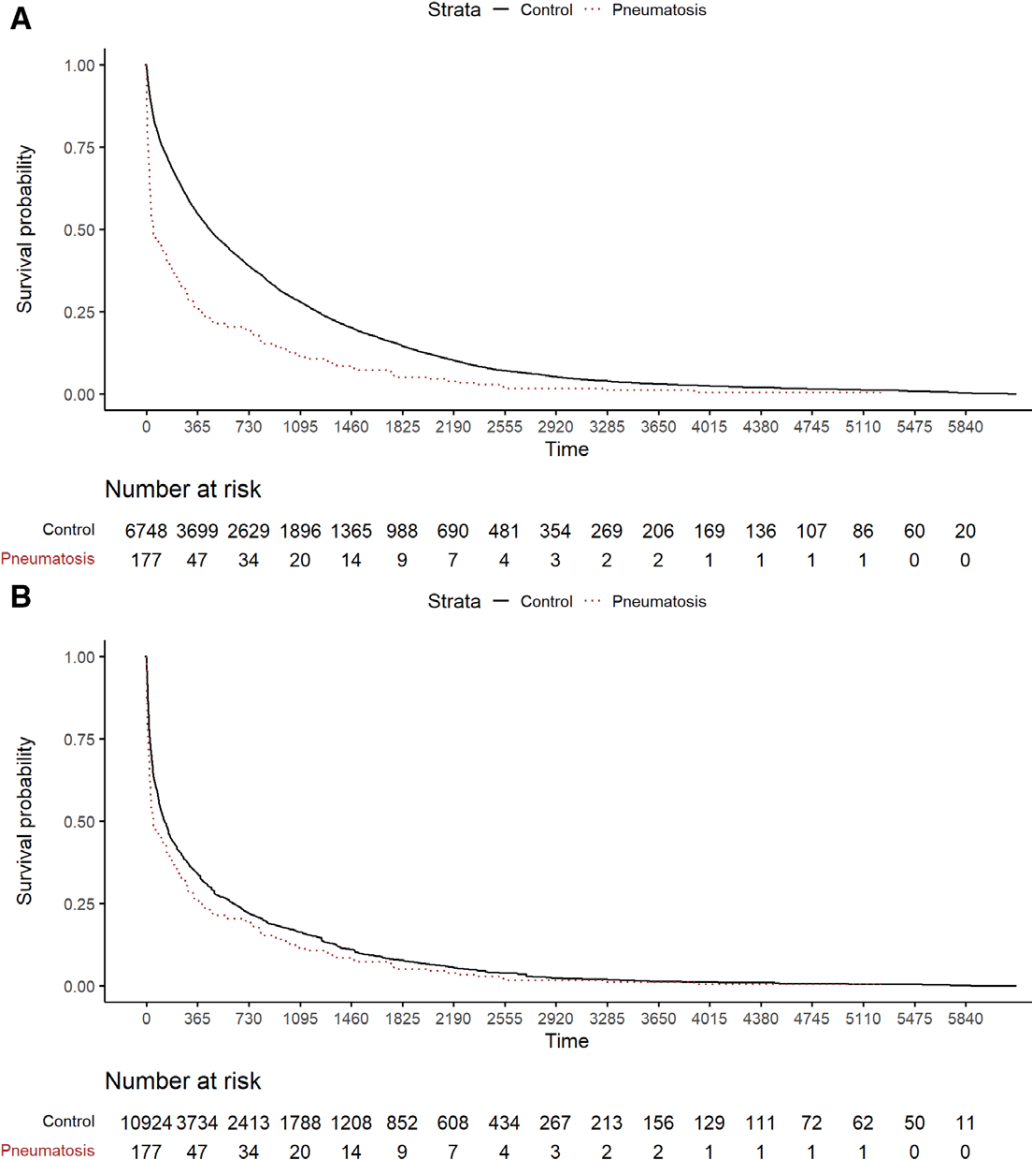
Cox regression for OS stratified by the presence or absence of pneumatosis intestinalis. A, Unadjusted model and (B) results following adjustment by the Elixhauser comorbidity variables. The *x* axis represents the number of days relative to the first time pneumatosis was diagnosed. Following adjustment, the association between pneumatosis on OS is statistically significant but substantially attenuated.

Cox regression of the effect of pneumatosis on OS in the unmatched, unadjusted data yielded a survival hazard ratio (HR) = 1.99; 95% CI, 1.88–2.10; *P* < 0.0001. The effect of pneumatosis on the HR was substantially attenuated when calculated in the matched cohort: HR = 1.24; 95% CI, 1.16–1.32; *P* = 0.021. Kaplan-Meier curves for OS stratified by the presence or absence of pneumatosis are shown in Figure 2. These curves show that when patients with pneumatosis are matched to similar patients without pneumatosis, survival is very similar.

Autopsy results were available for 6 patients who had a CT diagnosis of pneumatosis, did not undergo surgery, and then later died. One patient died the day of the imaging that demonstrated gastric pneumatosis from a gastric perforation due to a gastric fungal infection secondary to acute myeloid anemia and graft versus host disease. Two other patients had ischemic bowel and were not operated on because of end-stage cancer. The remaining 3 patients died 1 to 7 years after the pneumatosis diagnosis with no bowel disease noted at autopsy. One of these patients did undergo resection of the bowel segment that had pneumatosis but 3 years after that diagnosis was made.

## DISCUSSION

PI can be present with both benign and potentially lethal intra-abdominal pathologies. Previous series have emphasized the natural history of PI or factors that contribute to management decisions.^25^ Because PI results from disease processes such as malignancy, some of which themselves are associated with high mortality independent of bowel pathology, we examined a relatively long, overall 90-day mortality as our primary endpoint. We found that metastatic cancer, lymphoma, and liver disease were strong predictors of mortality. When adjusted for baseline medical conditions, we found that PI itself had little effect on mortality. When performing surgery on patients who had a diagnosis of PI, surgeons mostly cited indications consistent with major intra-abdominal pathology: the presence of abdominal pain, tenderness, or persistent sepsis. These observations suggest that PI itself was not the main indication for surgical intervention.

Prior studies have emphasized how PI might affect medical decision-making. For example, in one study of 305 patients with PI, a scoring system was developed to predict bowel ischemia or withdrawal of care based on the presence of PI. Lactate ≥ 2.0 mmol/L, age ≥ 70 years, tachycardia, and a neutrophil/lymphocyte ≥ 10.0% were associated with the need for surgical intervention.^25^ A positive score was 100% sensitive with a 64% specificity (ie, false positive rate = 36%). A similar multicenter study of 500 patients with data up to 2010 found that 40% of those with PI had either bowel ischemia or withdrawal of care. These events could be predicted with reasonable accuracy by a score consisting of serum lactate ≥ 2.0 mmol/L, hypotension, vasopressor dependency, and/or peritonitis.^26^ This observation was followed up with a 127-patient prospective study finding that serum lactate ≥ 2.0 mmol/L and the presence of peritonitis were most predictive of patients having bowel ischemia,^13^ an observation similar to other reports.^11,12,14^ These findings imply that decision-making to pursue laparotomy should be made by clinical features suggestive of bowel ischemia such as acidosis, peritonitis, or hypotension and not PI itself.

Prior studies examining mortality and PI found that sepsis,^16^ the presence of portal venous gas,^6,17^ peritonitis,^18^ and lack of bowel enhancement on CT examination^18^ were associated with mortality (Supplemental Table 4, see http://links.lww.com/AOSO/A355). Portal venous gas was inconsistently associated with mortality with at least one study not finding a relationship between it and mortality.^22^ None of the prior studies of PI and mortality had a control group. They were unable to examine the independent contribution of PI to mortality. We studied the contribution of PI to 90-day mortality by creating a control group of patients who also had unspecified gastrointestinal disorders. The PI group was compared with the control group by visual analysis of survival curves, full matching of all patients in the 2 groups, and logistic regression conditioned on risk factor variables. Taken together, these analyses suggested little to no contribution of PI itself to mortality. Rather, the underlying conditions associated with PI such as metastatic cancer, lymphoma, and liver disease were strongly associated with mortality. These findings suggest that when a patient with PI is evaluated, the decision to operate should be made on clinical features suggestive of an intra-abdominal source of sepsis such as peritonitis and that mortality will ultimately be related to the underlying cause of PI and not PI itself.

Had we only used a single methodology such as matching to assess the relationship between PI and mortality, we might have concluded a significant association existed because of a significant *P* value observed in the matched cohort. No such relationship was found with logistic regression. Visual assessment of the matched cohorts’ Kaplan-Meier survival curves showed how small the contribution of PI was to mortality. This sequence of analyses demonstrates the hazards of looking at data by a single statistical methodology, finding a statistically significant *P* value, and then concluding some factor contributes importantly to some outcome. The opposite can occur where a factor is important, but no association is found between the factor and outcome because the statistical test lacked sufficient power. When assessing the importance of a factor to an outcome, visual analysis can be very useful^27^ as is consideration of effect size.^28^ Some experts have advocated for only accepting results as important only if odds ratios are very large.^29^

The causes and pathogenesis of PI are unclear. A mechanical theory suggests that PI results from increased intraluminal pressure causing gas to dissect through the bowel wall.^3^ A bacterial theory proposes that gas-producing microbes translocate through the mucosa into the submucosa and produce gas in this layer. Several studies using gas chromatography methods demonstrated high partial pressures of hydrogen, which is not produced by human cells, within resected pneumatosis samples favoring a bacterial origin of the gas.^30,31^ A pulmonary theory suggests that PI may originate from the lungs, possibly from alveolar rupture in the presence of severe obstructive pulmonary disease (COPD) or mechanical ventilation. This allows gas to track into the retroperitoneum and mesenteric blood vessels,^32^ although this theory has been met with skepticism.^33–35^ There is currently no agreement in one theory over the other, and each remains grounded on limited evidence. Most cases of PI may represent a combination of these theories, where an initial insult results in permeability of the bowel wall, allowing the translocation of gas and possibly microorganisms through the mucosal layer. These potential pathophysiologic mechanisms are consistent with a benign origin of PI, such that its presence on imaging does not necessarily represent ischemic bowel or imminent death.

Our study has limitations. (1) There was missing data, as is often the case with most retrospective database studies. (2) Not all coding information was available for all the patients, limiting our ability to assess comorbidities within the full dataset. (3) Mortality was only known for patients who died at a UCLA facility. (4) Very few autopsies were performed for patients who died, limiting our ability to detect a misdiagnosis for patients who did not receive surgery. (5) The data were generated from a single health system such that medical decision-making might reflect local practice patterns that may differ from those in other health systems.

## CONCLUSIONS

Most patients with a diagnosis of PI survive without surgery. Of those who undergo surgery, nearly all have indications for laparotomy exclusive of PI. Mortality in patients who have PI is strongly associated with comorbid disease, with little to no independent association with PI. Our findings suggest that PI should not be a primary indication for surgical intervention.

## Supplementary Material


