# Supplementary material for: Association of Pneumatosis Intestinalis With Surgical Outcomes and Mortality: A Matched, Retrospective Cohort Study and Literature Review
Source: Ann Surg Open. 2024 Jun 21;5(3):e448. doi: 10.1097/AS9.0000000000000448 (PMC11415091; doi:10.1097/AS9.0000000000000448)
Supplement: Supplementary file 1 [file as9-5-e448-s001.pdf]

sTable 1. Elixhauser comorbidity scores for patients who died within 90 days of being diagnosed with pneumatosis intestinalis compared to those who survived. There are fewer patients in this analysis relative to the total population represented in Tabe 1 because of missing data to calculate Elixhauser scores.

	**Alive (N=194)**	**Dead (N=84)**	**SMD**
FLUID AND ELECTROLYTE DISORDERS	76 (39.2%)	54 (64.3%)	0.52
ALCOHOL ABUSE	16 (8.2%)	23 (27.4%)	0.52
LIVER DISEASE	63 (32.5%)	46 (54.8%)	0.46
CHF	23 (11.9%)	25 (29.8%)	0.45
WEIGHT LOSS	67 (34.5%)	45 (53.6%)	0.39
COAGULOPATHY	54 (27.8%)	37 (44.0%)	0.34
METASTATIC CANCER	14 (7.2%)	15 (17.9%)	0.33
ARRYTHMIA	39 (20.1%)	28 (33.3%)	0.3
RENAL FAILURE	54 (27.8%)	33 (39.3%)	0.24
LYMPHOMA	3 (1.5%)	5 (6.0%)	0.23
SOLID TUMOR, WITHOUT METASTASIS	31 (16.0%)	21 (25.0%)	0.22
UNCOMPLICATED DIABETES	11 (5.7%)	9 (10.7%)	0.18
OBESITY	11 (5.7%)	9 (10.7%)	0.18
DEFICIENCY ANAEMIA	15 (7.7%)	11 (13.1%)	0.18
PARALYSIS	2 (1.0%)	3 (3.6%)	0.17
AIDS/HIV	2 (1.0%)	0 (0%)	0.14
PUMONARY CIRCULATION DISORDERS	16 (8.2%)	10 (11.9%)	0.12
PERIPHERAL VASCULAR DISEASE	22 (11.3%)	13 (15.5%)	0.12
UNCOMPLICATED HTN	49 (25.3%)	17 (20.2%)	0.12
COMPLICATED HTN	42 (21.6%)	22 (26.2%)	0.11
COMPLICATED DIABETES	27 (13.9%)	15 (17.9%)	0.11
PSYCHOSES	5 (2.6%)	1 (1.2%)	0.1
DRUG ABUSE	6 (3.1%)	4 (4.8%)	0.086
DEPRESSION	31 (16.0%)	11 (13.1%)	0.082
HYPOTHYROIDISM	23 (11.9%)	12 (14.3%)	0.072
CHRONIC PULMONARY DISEASE	34 (17.5%)	16 (19.0%)	0.039
PEPTIC ULCER DISEASE, EXCLUDING BLEEDING	3 (1.5%)	1 (1.2%)	0.031
RHEUMATOID ARTHRITIS/COLLAGED VASCULAR DISEASE	19 (9.8%)	9 (10.7%)	0.03
VALVULAR DISEASE	17 (8.8%)	8 (9.5%)	0.026
OTHER NEUROLOGIC DISORDERS	20 (10.3%)	8 (9.5%)	0.026
BLOOD LOSS ANAEMIA	2 (1.0%)	1 (1.2%)	0.015
Mean Elixhauser Score(SD)	13.9 (11.2)	23.8 (9.3)	0.96

Small Effect SMD <=0.5 , Medium Effect 0.5-0.8, Large Effect SMD > 0.8

sTable 2. Complete Results of Logistic Regression

Characteristic	Odds Ratio	95% CI	p-value
Metastatic Cancer	4.78	4.23, 5.40	<0.001
Lymphoma	2.39	1.95, 2.94	<0.001
Liver Disease	2.26	2.10, 2.43	<0.001
Complicated HTN	1.91	1.73, 2.10	<0.001
Other Neurologic Disease	1.89	1.75, 2.05	<0.001
Paralysis	1.71	1.46, 2.00	<0.001
Coagulopathy	1.57	1.46, 1.70	<0.001
Peripheral Vascular Disease	1.52	1.41, 1.64	<0.001
Solid Tumor	1.46	1.34, 1.60	<0.001
Rheumatoid arthritis/CVD	1.45	1.30, 1.62	<0.001
Weight Loss	1.39	1.29, 1.50	<0.001
Renal Failure	1.38	1.27, 1.51	<0.001
Blood Loss Anemia	1.37	1.12, 1.67	0.002
Uncomplicated Diabetes	1.36	1.23, 1.50	<0.001
Drug Abuse	1.32	1.16, 1.52	<0.001
Complicated Diabetes	1.2	1.09, 1.32	<0.001
Sex (Male)	1.12	1.05, 1.20	<0.001
CHF	1.12	1.03, 1.23	0.011
Time First Pneumatosis Dx	1	1.00, 1.00	<0.001
Obesity	0.89	0.79, 1.01	0.071
Uncomplicated HTN	0.87	0.81, 0.94	<0.001
Pulmonary Circulation Disorders	0.84	0.76, 0.94	0.002
Chronic Pulmonary Disease	0.71	0.65, 0.77	<0.001
AIDS	0.67	0.46, 0.97	0.035
Alcohol Abuse	0.66	0.59, 0.73	<0.001
Peptic Ulcer Disease	0.5	0.42, 0.61	<0.001
Psychoses	0.17	0.13, 0.21	<0.001
Cardiac Arrythmias	1.03	0.96, 1.11	0.4
Depression	1.01	0.93, 1.11	0.7
Age	1	1.00, 1.00	0.4
Deficiency Anemia	0.98	0.89, 1.08	0.7
Valvular Disease	0.97	0.88, 1.08	0.6
Hypothyroidism	0.97	0.88, 1.07	0.5
Pneumatosis	0.3	0.01, 18.6	0.6
PNEUM * AGE	0.99	0.97, 1.01	0.2
PNEUM * SEX			
PNEUM * Male	0.86	0.44, 1.66	0.6
PNEUM * chf	3.35	1.31, 9.17	0.014
PNEUM * carit	0.84	0.43, 1.64	0.6
PNEUM * valv	1.14	0.44, 3.03	0.8

PNEUM * pcd	1.01	0.36, 2.93	>0.9
PNEUM * pvd	1.46	0.70, 3.15	0.3
PNEUM * hypunc	1	0.58, 2.21	0.7
PNEUM * hypc	0.37	0.14, 0.94	0.037
PNEUM * para	148,667	56.0, NA	0.9
PNEUM * ond	0.56	0.25, 1.25	0.2
PNEUM * cpd	3.44	1.52, 8.10	0.004
PNEUM * diabunc	1.54	0.55, 4.59	0.4
PNEUM * diabc	0.76	0.30, 1.94	0.6
PNEUM * hypothy	2.18	0.82, 6.17	0.13
PNEUM * rf	1.65	0.73, 3.80	0.2
PNEUM * ld	0.78	0.35, 1.76	0.5
PNEUM * pud	0.4	0.04, 5.40	0.5
PNEUM * aids	0		
>0.9			
PNEUM * lymph	2.42	0.21, 70.5	0.5
PNEUM * metacanc	1.56	0.43, 6.79	0.5
PNEUM * solidtum	1.64	0.69, 3.99	0.3
PNEUM * rheumd	1.94	0.67, 6.09	0.2
PNEUM * coag	0.79	0.35, 1.76	0.6
PNEUM * obes	0.67	0.20, 2.27	0.5
PNEUM * wloss	0.65	0.32, 1.31	0.2
PNEUM * blane	1.66	0.18, 38.0	0.7
PNEUM * dane	0.84	0.31, 2.32	0.7
PNEUM * alcohol	5.1	1.74, 16.3	0.004
PNEUM * drug	0.55	0.14, 2.34	0.4
PNEUM * psycho	31.3	3.38, 749	0.007
PNEUM * depre	2.33	0.94, 6.08	0.075
PNEUM * ResTime	1	1.00, 1.00	0.3

1 OR = Odds Ratio, CI = Confidence Interval

- chf, for congestive heart failure;
- carit, for cardiac arrhythmias;
- valv, for valvular disease;
- pcd, for pulmonary circulation disorders;
- pvd, for peripheral vascular disorders;
- hypunc, for hypertension, uncomplicated;
- hypc, for hypertension, complicated;
- para, for paralysis;
- ond, for other neurological disorders;
- cpd, for chronic pulmonary disease;
- diabunc, for diabetes, uncomplicated;
- diabc, for diabetes, complicated;
- hypothy, for hypothyroidism;

- rf, for renal failure;
- ld, for liver disease;
- pud, for peptic ulcer disease, excluding bleeding;
- aids, for AIDS/HIV;
- lymph, for lymphoma;
- metacanc, for metastatic cancer;
- solidtum, for solid tumor, without metastasis;
- rheumd, for rheumatoid arthritis/collaged vascular disease;
- coag, for coagulopathy;
- obes, for obesity;
- wloss, for weight loss;
- fed, for fluid and electrolyte disorders;
- blane, for blood loss anemia;
- dane, for deficiency anemia;
- alcohol, for alcohol abuse;
- drug, for drug abuse;
- psycho, for psychoses;
- depre, for depression;

sTable 3. Test fit of different match models and statistics for full match. Below this table are summary data for baseline characteristics of the 287 pneumatosis and 21,164 control patients entered into the matched cohort analysis.

Method	Matched	Unmatched	Distance
Unbalanced			0.0128
nearest	287	0	0.0144
optimal	287	0	0.0144
full	287	0	0.0019
cem	26	261	
exact		No Match found	

```
Summary of Balance for All Data:
          Means Treated Means Control Std. Mean Diff. Var. Ratio eCDF Mean eCDF Max
distance         0.0593        0.0128          0.7298     6.9716    0.3327   0.5506
AGE             60.7003       65.1576         -0.2504     1.0988    0.0482   0.1616
SEXFemale        0.4425        0.5638         -0.2443          .    0.1213   0.1213
SEXMale          0.5575        0.4362          0.2443          .    0.1213   0.1213
chf              0.2195        0.0853          0.3242          .    0.1342   0.1342
carit            0.4321        0.1426          0.5844          .    0.2895   0.2895
valv             0.1463        0.0602          0.2437          .    0.0861   0.0861
pcd              0.1150        0.0441          0.2221          .    0.0709   0.0709
pvd              0.2997        0.0741          0.4924          .    0.2256   0.2256
hypunc           0.3798        0.2047          0.3608          .    0.1751   0.1751
hypc             0.2439        0.1067          0.3194          .    0.1372   0.1372
para             0.0348        0.0117          0.1264          .    0.0232   0.0232
ond              0.2509        0.0721          0.4124          .    0.1788   0.1788
cpd              0.2021        0.0954          0.2658          .    0.1067   0.1067
diabunc          0.1289        0.0770          0.1549          .    0.0519   0.0519
diabc            0.1777        0.0747          0.2696          .    0.1030   0.1030
hypothy          0.1254        0.0881          0.1127          .    0.0373   0.0373
rf               0.3868        0.1369          0.5131          .    0.2499   0.2499
ld               0.4808        0.1453          0.6716          .    0.3355   0.3355
pud              0.0279        0.0154          0.0758          .    0.0125   0.0125
aids             0.0070        0.0075         -0.0060          .    0.0005   0.0005
lymph            0.0279        0.0200          0.0476          .    0.0078   0.0078
metacanc         0.1220        0.1031          0.0576          .    0.0189   0.0189
solidtum         0.2474        0.3132         -0.1525          .    0.0658   0.0658
rheumd           0.1010        0.0484          0.1747          .    0.0527   0.0527
coag             0.4739        0.0986          0.7516          .    0.3753   0.3753
obes             0.0767        0.0320          0.1679          .    0.0447   0.0447
wloss            0.4495        0.1372          0.6277          .    0.3123   0.3123
blane            0.0244        0.0235          0.0059          .    0.0009   0.0009
dane             0.1254        0.0605          0.1960          .    0.0649   0.0649
alcohol          0.1568        0.0289          0.3517          .    0.1279   0.1279
drug             0.0557        0.0336          0.0964          .    0.0221   0.0221
psycho           0.0209        0.0080          0.0900          .    0.0129   0.0129
depre            0.1777        0.1039          0.1931          .    0.0738   0.0738
score           23.0000        8.6861          1.0629     1.5093    0.1959   0.4701
ResTime      17328.0662    17275.3670          0.0338     0.9175    0.0192   0.0591

Summary of Balance for Matched Data:
          Means Treated Means Control Std. Mean Diff. Var. Ratio eCDF Mean eCDF Max
distance         0.0593        0.0595         -0.0030     0.9652    0.0012   0.0209
AGE             60.7003       61.4381         -0.0414     1.1114    0.0150   0.0529
```

SEXFemale	0.4425	0.4241	0.0370	.	0.0184	0.0184
SEXMale	0.5575	0.5759	-0.0370	.	0.0184	0.0184
chf	0.2195	0.2093	0.0246	.	0.0102	0.0102
carit	0.4321	0.4214	0.0215	.	0.0107	0.0107
valv	0.1463	0.1462	0.0005	.	0.0002	0.0002
pcd	0.1150	0.1042	0.0338	.	0.0108	0.0108
pvd	0.2997	0.2845	0.0332	.	0.0152	0.0152
hypunc	0.3798	0.3858	-0.0125	.	0.0061	0.0061
hypc	0.2439	0.2272	0.0389	.	0.0167	0.0167
para	0.0348	0.0380	-0.0171	.	0.0031	0.0031
ond	0.2509	0.2577	-0.0157	.	0.0068	0.0068
cpd	0.2021	0.2111	-0.0225	.	0.0090	0.0090
diabunc	0.1289	0.1281	0.0023	.	0.0008	0.0008
diabc	0.1777	0.1885	-0.0283	.	0.0108	0.0108
hypothy	0.1254	0.1221	0.0102	.	0.0034	0.0034
rf	0.3868	0.3788	0.0164	.	0.0080	0.0080
ld	0.4808	0.4929	-0.0241	.	0.0120	0.0120
pud	0.0279	0.0345	-0.0400	.	0.0066	0.0066
aids	0.0070	0.0140	-0.0842	.	0.0070	0.0070
lymph	0.0279	0.0296	-0.0105	.	0.0017	0.0017
metacanc	0.1220	0.1404	-0.0565	.	0.0185	0.0185
solidtum	0.2474	0.2662	-0.0436	.	0.0188	0.0188
rheumd	0.1010	0.0973	0.0125	.	0.0038	0.0038
coag	0.4739	0.4503	0.0471	.	0.0235	0.0235
obes	0.0767	0.0751	0.0060	.	0.0016	0.0016
wloss	0.4495	0.4377	0.0237	.	0.0118	0.0118
blane	0.0244	0.0313	-0.0446	.	0.0069	0.0069
dane	0.1254	0.1150	0.0316	.	0.0105	0.0105
alcohol	0.1568	0.1603	-0.0095	.	0.0035	0.0035
drug	0.0557	0.0568	-0.0046	.	0.0011	0.0011
psycho	0.0209	0.0192	0.0117	.	0.0017	0.0017
depre	0.1777	0.1869	-0.0240	.	0.0092	0.0092
score	23.0000	23.2511	-0.0186	0.8634	0.0159	0.0552
ResTime	17328.0662	17306.4096	0.0139	0.8350	0.0292	0.0812

	Std. Pair Dist.
distance	0.0019
AGE	1.0126
SEXFemale	0.7798
SEXMale	0.7798
chf	0.4048
carit	0.4158
valv	0.3242
pcd	0.4272
pvd	0.2604
hypunc	0.6907
hypc	0.4944
para	0.1311
ond	0.2780
cpd	0.4619
diabunc	0.4340
diabc	0.3713
hypothy	0.4782
rf	0.4314
ld	0.3444
pud	0.1972
aids	0.1278
lymph	0.1906
metacanc	0.4599
solidtum	0.8709
rheumd	0.4919
coag	0.2119
obes	0.2149
wloss	0.3963

```
blane              0.3461
dane               0.2690
alcohol            0.1249
drug               0.2982
psycho             0.1846
depre              0.4859
score              0.4858
ResTime            1.1718

Sample Sizes:
              Control Treated
All           21164.      287
Matched (ESS)  1520.02    287
Matched       21164.      287
Unmatched         0.        0
Discarded         0.        0
```

sTable 4. Literature Review including prior reports of 40 or more patients with pneumatosis intestinalis.

Author	Year	Reference Number	n	Conclusion
Ohmiya	2022	20	167	Small bowel pneumatosis associated with complications and poor prognosis
DuBose	2013	26	500	60% can be managed nonoperatively
Lee	2014	18	123	Most common cause of PI was mesenteric ischemia. Peritonitis and decreased or bowel wall enhancement were associated with mortality
Ferrada	2017	13	127	Exploration recommended for elevated lactate and peritonitis
Graber	2022	15	109	Patients referred for mesenteric ischemia pneumatosis of the colon, elevated AST and perfusion inhomogeneities predict mortality
Treyaud	2016	22	149	Ischemia caused 54% of pneumatosis cases
Goyal	2017	14	167	Benign cause for pneumatosis in 58%. Predictors for worrisome disease include serum lactate, underlying bowel disease and bowel obstruction.
Ferrada	2017	13	127	Benign disease in 62%. Surgical exploration recommended for patients with elevated lactate and/or peritonitis.
Lee	2014	18	123	Developed a risk score to predict mortality based on signs of peritoneal irritation and decreased bowel enhancement on CT
Lee	2013	19	84	Benign disease in 56%. Worrisome features include pneumatosis of the small bowel, mesenteric stranding, bowel wall thickening, and ascites.
Bani	2013	11	256	Bowel ischemia/necrosis associated with older age, peritoneal signs, and high BUN. CT findings alone do not reliably predict the need for surgery.
Shinagare	2012	21	48	No symptoms associated with pneumatosis in 71%. Pneumatosis associated with molecular targeted therapy for cancer.
Duron	2011	12	150	Benign disease in 36%. Abnormal physical examination findings were more predictive of the need for surgical intervention than laboratory values or radiographic findings.
Wayne	2010	23	88	Developed score predictive of bowel ischemia based on abdominal pain, elevated lactate, pneumatosis in small bowel and vascular disease score

Lassandro	2010	17	102	Portal vein gas associated with bowel infarction. Infarction present in 52% of cases
Greensteen	2007	16	40	Surgery necessary in 35% of cases. Need for surgery associated with age > 60, elevated WBC and emesis. Sepsis associated with mortality

sFigure 1. Graphic of full match for age, sex and date of the first radiograph demonstrating pneumatosis. The x-axis for the date plot is the number of days between 1/1/1970 and the date of the radiograph.

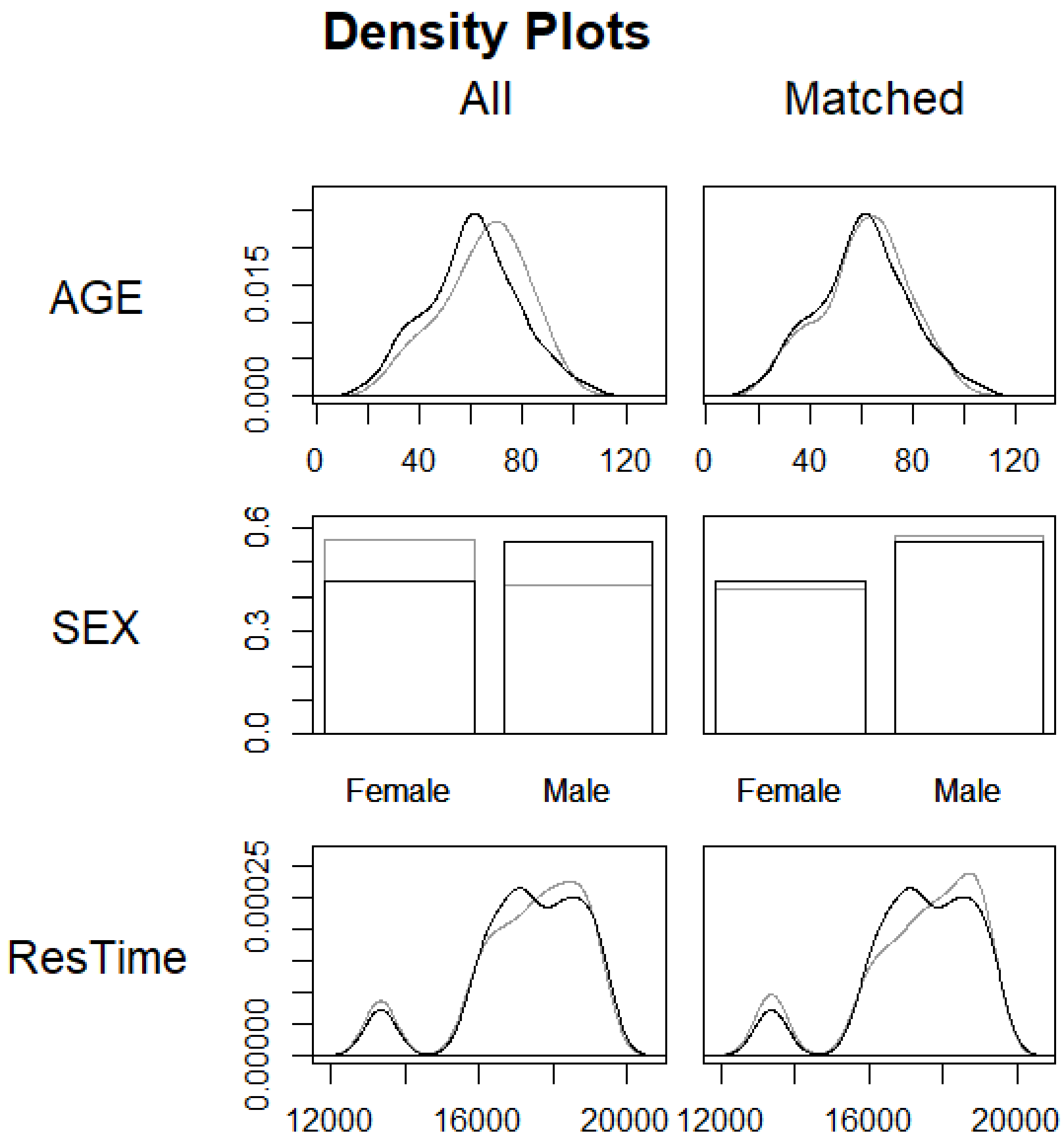

R code for Pneumatosis Analysis

```
# Pneuma3 - corrections following manual verification of classifications resulting in n=315 not 316
 # TimeToSurg_Kyle_OpFindings EL CORRECTIONS 110423.xlsx is file with corrected classifications

 library(tidyverse)
 library(tidytext)
 library(data.table)
 library(stringi)

# pacman:: to load libraries (can remove and update packages)
pacman::p_load(tidyverse,tidytext,data.table,stringi)


 setwd("C:\\Users\\ehlsu\\OneDrive\\Research\\Pneumatosis intestinalis")
 setwd('F:\\Inbound\\CTSI\\ELivingston_23_23-001036\\Data')
 setwd('F:\\Inbound\\CTSI\\ELivingston_23_23-001036\\Analysis')
 df <- read.csv("Imaging_Narratives.csv")     #7,431,222 lines for information on 16,728 patients in ULEAD
 length(unique(df$IP_PATIENT_ID)  #n=16,728 patients
 length(unique(df$IP_ORDER_PROC_ID)  #n=425,832 exams

 ##############################
 1. Import Imaging_Narratives.csv from ULEAD into df-this file has multiple lines for each record (n=16,728 patients)
 2. Find rows that contain the words pneumatosis of pneumotosis.
        i. Convert to all lower case
             ii. Use grepl and filter to find rows containing the words pneumatosis etc. (n=1397 lines with the words)
             iii. Save as LinesWithPneum.csv-use this for patient and study ID numbers for records of interest
 3. Create file with each image by order# that has the words and recreate the entire paragraph
             i. Pull all lines from Imaging_Narratives.csv with the order numbers from LinesWithPneum.csv using inner_join (n=1260 orders)
             ii. Create dataframe with only the order numbers for joining using subset-then join the datafrframes
             ii. reassemble individual lines into paragraphs using stri_c (result in df3)
 4. Break paragraphs into individual full sentences using RegEx identification of periods as the end of a sentences (df4)
 5. Find positive and negative wording in the sentences after filtering for sentences that have pneum in them
             i. df5 is working file with sentences
        ii. polarity.csv is file that has the pos/neg classification
        iii. Combine Pat and Order ID with pos/neg (polarity) and full paragraph: AllInfoClassified.csv
        iiii. NEXT TIME CHANGE GREPL MATCH TO \bno\b and \bcant\b TO AVOID PICKING THESE UP IN THE MIDDLE OF WORDS




#References
#Join dataframes based on all instances of one with ID numbers from another: https://stackoverflow.com/questions/68702694/how-can-i-keep-data-from-one-dataframe-based-on-its-appearance-in-a-second-dataf
#Subsetting: https://sparkbyexamples.com/r-programming/r-subset-data-frame-with-examples/#:~:text=If%20you%20wanted%20to%20get,variables)%20from%20the%20data%20frame.
#Recreate paragraph: https://stackoverflow.com/questions/9944816/unique-on-a-dataframe-with-only-selected-columns
```

```
#Recreate paragraph: https://stackoverflow.com/questions/61463160/how-to-concatenate-rows-based-on-group-as-quickly-as-possible
#Find negative sentences: https://stackoverflow.com/questions/47531852/how-to-detect-negative-sentences-with-sentimentr-or-qdap
#Filter (match) df on vector:  https://stackoverflow.com/questions/9350025/filtering-a-data-frame-on-a-vector
#Excel macro to highlight words in excel: https://www.indeed.com/career-advice/career-development/how-to-highlight-words-in-excel#:~:text=After%20you%20press%20%22F5%2C%22,that%20contains%20your%20chosen%20words.
    and https://www.extendoffice.com/documents/excel/3775-excel-highlight-part-of-text-in-cell.html  NOTE HIGHLIGHT IS ONLY IN CELLS THEMSELVES_NOT IN PREVIEW BOX AT TOP OF SHEET
#Identify end of sentnce by period using RegEx https://stackoverflow.com/questions/47211643/tokenizing-sentences-with-unnest-tokens-ignoring-abbreviations
# NLP for medical terminology: https://amunategui.github.io/speak-like-a-doctor/index.html
# Test NLP/RegEx phrases against text: https://regex101.com/
# Using PACMAN for package management https://statisticsglobe.com/pacman-r-package
# Join v Merge: https://stackoverflow.com/questions/1299871/how-to-join-merge-data-frames-inner-outer-left-right



########################          Working in ULEAD

############ Find lines with sought after words

 df$NARRATIVE <- tolower(df$NARRATIVE)   # make all lower case

  df1 <- df %>% filter(grepl('pneumatosis | pneumotosis',df$NARRATIVE))  #n=1397 lines of text with the words of interest

  setwd('F:\\Inbound\\CTSI\\ELivingston_23_23-001036\\Analysis')
  write.csv(df1,"LinesWithPneum.csv")      #File with lines from imaging narrative that has Pneumatosis words-use for Patient and Order ID

#####################################################

############   Recreate full paragraph of reports for each imaging order in LinesWithPneum.csv


ID <- subset(df1,select='IP_ORDER_PROC_ID') #Only the column with order numbers for joining
df2<- df %>% inner_join(ID, by="IP_ORDER_PROC_ID") #Keep all instances of df that have order#'s in df1
 length(unique(df2$IP_ORDER_PROC_ID))  #n=1260
 length(unique(df1$IP_ORDER_PROC_ID))  #n=1260 unique image orders with pneum.

setDT(df2)                              #Convert to datatable
setkey(df2, IP_ORDER_PROC_ID,LINE)
df3 <- df2[, .(NARRATIVE = stri_c(NARRATIVE, collapse = " ")), by = IP_ORDER_PROC_ID]
#collapses individual lines into a paragraph

#######################################


#####################################################

########### Break back into individual sentences
```

```
df4 <- unnest_tokens(df3, input = "NARRATIVE", output = "Sentence", token = "regex",
              pattern = "(?<!\\b\\p{L}r)\\.")
#Break into sentence using RegEx identification of periods



##########################################################

########### Find negative/positive language

 df5 <- df4 %>% filter(grepl('pneumatosis | pneumotosis',df4$Sentence))  #n=1682 lines
of text with the words of interest

polarity <- data.frame(df5, pol = NA)
polarity$pol <- ifelse(grepl(paste(lexicon::hash_valence_shifters[y==1]$x,collapse =
'|'), tolower(df5$Sentence)),'Negative','Positive')
write.csv(polarity,"PosNegPneumClassification.csv")    #write file for manual review
of classification
length(unique(polarity$IP_ORDER_PROC_ID))   n=1260  ordered images

########################################################

###########  Add patient ID and full paragraph back to polarity file

PatOrderID <- subset(df1,select=c('IP_ORDER_PROC_ID','IP_PATIENT_ID'))
PolOrderID <- subset(polarity,select=c('IP_ORDER_PROC_ID','pol'))

df7 <- df3 %>% inner_join(PatOrderID,by='IP_ORDER_PROC_ID')
df7 <- df7 %>% inner_join(PolOrderID,by='IP_ORDER_PROC_ID')
df8 <- df7[!duplicated(df7[,c('IP_ORDER_PROC_ID')]),]

write.csv(df8,"AllInfoClassified.csv")

#### Numerical Checks

 length(unique(df7$IP_ORDER_PROC_ID))  #n=1260
 length(unique(df7$IP_PATIENT_ID))  #n=766 unique patients.

 dfNeg <- df7[which(df7$pol=='Negative'),]   #n=3545
 dfPos <- df7[which(df7$pol=='Positive'),]   #n=5293
 dfPosUniquePt <- unique(dfPos$IP_PATIENT_ID)   #get unique ids of the patients in the
df  n=290 unique patients
 dfNegUniquePt <- unique(dfNeg$IP_PATIENT_ID)   #get unique ids of the patients in the
df  n=607 unique patients


#  PatIDNeg <-  dfNeg[!duplicated(dfNeg[,c('IP_PATIENT_ID')]),]   #deduplicate Pat ID
numbers
#   PatIDPos <-  dfPos[!duplicated(dfPos[,c('IP_PATIENT_ID')]),]   #deduplicate

#####################################################################################
##########################

###########    CREATE FILES WITH PATIENTS WHO HAVE AND DO NOT HAVE PNEUMATOSIS FROM
HAND REVIEWED CLASSIFICATION

dfPos <- readxl::read_xlsx("PosNegPneumClassification2Dedup.xlsx",sheet='Pos')  # read
in order numbers for images that have pneumatosis
dfNeg <- readxl::read_xlsx("PosNegPneumClassification2Dedup.xlsx",sheet='Neg')  # read
in order numbers for images that do not have pneumatosis
dfAllInfo <- read.csv("AllInfoClassified2.csv") # read in patient and order numbers
PatID <- subset(dfAllInfo,select=c('IP_ORDER_PROC_ID','IP_PATIENT_ID'))    #Creat file
with order IDs matched to Pat IDs
```

```
dfPos <- dfPos %>% inner_join(PatID,by='IP_ORDER_PROC_ID')   # Add Pat ID to fdPos
file matched on order numbers
dfNeg <- dfNeg %>% inner_join(PatID,by='IP_ORDER_PROC_ID')

length(unique(dfPos$IP_PATIENT_ID))   # n= 316 positive patients
length(unique(dfNeg$IP_PATIENT_ID))   # n= 548  Negative patients

length(unique(dfPos$IP_ORDER_PROC_ID))   # n= 621 positive studies
length(unique(dfNeg$IP_ORDER_PROC_ID))   # n= 696 negative studies

write.csv(dfPos,"PosPneumPatID.csv")     #Create files with ID and order number for
Definitelyh Positive Patients
write.csv(dfNeg,"NegPneumPatID.csv")

############  GET DATE/TIME FOR RADIOLOGY STUDIES

df <- read.csv(('F:\\Inbound\\CTSI\\ELivingston_23_23-001036\\Data\\Imaging.csv')
#Read Imaging files
ImageDate <- subset(df,select=c('IP_ORDER_PROC_ID','RESULT_TIME'))    #Create file
with order IDs matched to Pat IDs
df1 <- read.csv('F:\\Inbound\\CTSI\\ELivingston_23_23-
001036\\Data\\PosPneumPatID.csv')  #Read in PosPneum file
dfImageDate <- df1 %>% inner_join(ImageDate,by='IP_ORDER_PROC_ID')   # Add Pat ID t
write.csv(dfImageDate,'F:\\Inbound\\CTSI\\ELivingston_23_23-
001036\\Analysis\\PosPneumPatID.csv')

######################################################################################
####################################3

##############  EXTRACT PATHOLOGY FILES FOR PNEUM PATIENTS

 setwd('F:\\Inbound\\CTSI\\ELivingston_23_23-001036\\Data')
 setwd('F:\\Inbound\\CTSI\\ELivingston_23_23-001036\\Analysis')

df <- read.csv("Pathology_Results.csv")
dfPos <- read.csv("PosPneumPatID.csv")
PatID <- subset(dfPos,select=c('IP_ORDER_PROC_ID','IP_PATIENT_ID'))    #Create file
with order IDs matched to Pat IDs
PatID <- as.data.frame(unique(PatID$IP_PATIENT_ID))
#Get only the unique patient IDs (n=316)
PatID <- PatID %>% rename("IP_PATIENT_ID"="unique(PatID$IP_PATIENT_ID)")
dfPath <- PatID %>% inner_join(df,by='IP_PATIENT_ID')   # Add path results to patID
file

df1 <- read.csv("F:\\Inbound\\CTSI\\ELivingston_23_23-001036\\Data\\Pathology.csv")
#Read Pathology file to get dates
PathNum <- subset(df1,select=c('IP_ORDER_PROC_ID','ORDER_TIME'))                  #
Get order number date from Pathology file (result time has many missing values)
dfPath <- dfPath %>% inner_join(PathNum,by='IP_ORDER_PROC_ID')   # Add date to path
results


setDT(dfPath)                               #Collapse RESULT_TXT to a single
paragraph
setkey(dfPath, IP_ORDER_PROC_ID,SORT_KEY)
dfPathPar <- dfPath[, .(RESULT_TXT = stri_c(RESULT_TXT, collapse = " ")), by =
IP_ORDER_PROC_ID]  #collapses individual lines into a paragraph

dfPathNoText <- subset(dfPath,select= -c(RESULT_TXT))                        #Remove 1
line text from main file
```

```
dfPathNoText <- distinct(dfPathNoText,IP_ORDER_PROC_ID, .keep_all=TRUE)      #
DeDuplicate main file
dfFinal <- dfPathNoText %>% inner_join(dfPathPar,by='IP_ORDER_PROC_ID')   # Add date
to full text to pathology data

 setwd('F:\\Inbound\\CTSI\\ELivingston_23_23-001036\\Analysis')
 write.csv(dfFinal,"PneumPathResults.csv")                               #Working
path file (n=4506 records, 285 unique patients)


#####################################################################################
########################################

 ###################################         KEEP PATHOLOGY FILES MORE RECENT THAN DAY
OF RADIOLOGY IMAGE

 dfRad <- read.csv("PosPneumPatID.csv")
 dfPath <- read.csv("PneumPathResults.csv")

 dfRad$RESULT_TIME <- as.Date(dfRad$RESULT_TIME,format = "%m/%d/%Y")   #Convert to
date formsat
 dfPath$ORDER_TIME <- as.Date(dfPath$ORDER_TIME,format = "%m/%d/%Y")

 dfRadFirstDate <- dfRad %>% group_by(IP_PATIENT_ID) %>%               #Cut data for
first radiology date for any patient only (n=316 patients)
     slice(which.min(RESULT_TIME))

dfRadFirstDate  <- subset(dfRadFirstDate, select=c(IP_PATIENT_ID,RESULT_TIME))  #keep
only patient ID and first radiology image date
dfPath <- dfPath %>% inner_join(dfRadFirstDate, by="IP_PATIENT_ID") #Add first
radiology date to path file (RESULT_TIME is for radiology, ORDER_TIME for path)

dfPath <- subset(dfPath, ORDER_TIME > RESULT_TIME)    #Keeps only path entries
occuring after first radiology entry (n=2816)
dfPath$Days <- dfPath$ORDER_TIME-dfPath$RESULT_TIME       #Number of days between path
(ORDER_TIME) and first raiology date (RESULT_TIME)
dfPath <- subset(dfPath, dfPath$Days < 30)                 #Only keep path results
that are within 30 days of the radiology report (n=200)

write.csv(dfPath,"PathAfterFirstRadiology.csv")

#####################################################################################
##########################################


############# NLP pathology file - IDENTIFY KEYWORDS IN NARRATIVE REPORT


dfPath <-
readxl::read_xlsx("PathAfterFirstRadiology.xlsx",sheet='PathAfterFirstRadiology')
dfKW <- readxl::read_xlsx("PathAfterFirstRadiology.xlsx",sheet='Key Words')
str()
########   CLEAN PATH READS

Text_To_Clean_Sentences <- function(text_blob) {
  # swap all sentence ends with code 'ootoo'
  text_blob <- gsub(pattern=';|\\.|!|\\?', x=text_blob, replacement='ootoo')

  # remove all non-alpha text (numbers etc)
  text_blob <- gsub(pattern="[^[:alpha:]]", x=text_blob, replacement = ' ')

  # force all characters to lower case
  text_blob <- tolower(text_blob)
```

```
  # remove any small words {size} or {min,max}
  text_blob <- gsub(pattern="\\W*\\b\\w{1,2}\\b", x=text_blob, replacement=' ')

  # remove contiguous spaces
  text_blob <- gsub(pattern="\\s+", x=text_blob, replacement=' ')

  # split sentences by split code
  #sentence_vector <- unlist(strsplit(x=text_blob, split='ootoo',fixed = TRUE))
  sentence_vector <- text_blob
   return (sentence_vector)
}

Trim <- function( x ) {
  # http://stackoverflow.com/questions/2261079/how-to-trim-leading-and-trailing-
whitespace-in-r
  gsub("(^[[:space:]]+|[[:space:]]+$)", "", x)
}

dfPathProc <- dfPath
dfPathProc$RESULT_TXT <- Text_To_Clean_Sentences(dfPathProc$RESULT_TXT)
dfPathProc$RESULT_TXT <- Trim(dfPathProc$RESULT_TXT)

###### CLASSIFY TEXT BY KEYWORDS

########### Find negative/positive language

words <- paste(pull(dfKW),collapse = '|')      #Need to use PULL to convert a tibble
to char vector
polarity <- data.frame(dfPathProc, pol = NA)
polarity$pol <- ifelse(grepl(words,
tolower(polarity$RESULT_TXT)),'Positive','Negative')

write.csv(polarity,"PathClassification.csv")    #write file for manual review of
classification
length(unique(polarity$IP_ORDER_PROC_ID))   n=1260  ordered images

#Test matching: https://regex101.com/

##########################################################

################  BUILD/UPDATE MASTER FILE]
df <- read.csv("MasterKnownPneum.csv")
df2 <- read.csv("ALLInfoClassified2.csv")
df3 <- read.csv("PathAfterFirstRadiology.csv")
df4 <- read.csv("PathClassification.csv")

df1 <- subset(df,select=c("IP_PATIENT_ID","IP_ORDER_PROC_ID","CT_RESULT_TIME"))
df1 <- df1 %>% rename("RAD_ORDER_ID"="IP_ORDER_PROC_ID")
df1 <- df1 %>% rename("RAD_DATE"="CT_RESULT_TIME")
df1$RAD_DATE <- as.Date(df1$RAD_DATE,format = "%m/%d/%Y")

df2 <- subset(df2,select=c(IP_ORDER_PROC_ID,NARRATIVE))
df2 <- df2 %>% rename("RAD_ORDER_ID"="IP_ORDER_PROC_ID")
df2 <- df1 %>% inner_join(df2, by="RAD_ORDER_ID")
df2 <- df2 %>% rename("RAD_TEXT"="NARRATIVE")
df2 <- df2[order(df2$IP_PATIENT_ID,df2$RAD_DATE),]  #Pneum file for master-sorted

df3 <-
subset(df3,select=c("IP_PATIENT_ID","IP_ORDER_PROC_ID","RESULT_TXT","RESULT_TIME","Day
s"))
df3 <- df3 %>% rename("PATH_ORDER_ID"="IP_ORDER_PROC_ID")
df3 <- df3 %>% rename("PATH_TXT"="RESULT_TXT")
```

```
df3 <- df3 %>% rename("PATH_DATE"="RESULT_TIME")
df3 <- df3 %>% rename("DAYS_RAD_PATH"="Days")       #Path file

df4 <- subset(df4,select=c("PATH_ORDER_ID","PATH_CLASSIFICATION"))
df3 <- df3 %>% inner_join(df4,by="PATH_ORDER_ID")   #add classification to path file

dfx <- df2 %>% left_join(df3,by="IP_PATIENT_ID")  #Final Master file
dfy <- merge(df2,df3,by="IP_PATIENT_ID", all.x = TRUE)  #Merge keeps all rows on left
and right


length(unique(dfx$IP_PATIENT_ID))
length(unique(df2$IP_PATIENT_ID))
length(unique(df3$IP_PATIENT_ID))
length(unique(dfy$IP_PATIENT_ID))
length(unique(dfy$PATH_ORDER_ID))

write.csv(dfx,"MasterRadPath.csv")
write.csv(dfy,"MasterRadPath2.csv")

######################################################

##############  GET PROCEDURE FILES FOR PNEUM Patients

setwd('F:\\Inbound\\CTSI\\ELivingston_23_23-001036\\Data')
df <- read.csv("Procedures.csv")
dfID <- read.csv("PosPneumPatID.csv")
dfID <- subset(dfID,select=c('IP_PATIENT_ID','RESULT_TIME'))
dfPneumProc <- dfID %>%  inner_join(df,by="IP_PATIENT_ID")
dfPneumProc <- dfPneumProc %>% rename("RAD_DATE"="RESULT_TIME")
dfPneumProc$RAD_DATE <- as.Date(dfPneumProc$RAD_DATE,format = "%m/%d/%Y")
dfPneumProc$PROCEDURE_DATE <- as.Date(dfPneumProc$PROCEDURE_DATE,format = "%m/%d/%Y")
dfPneumProc <- subset(dfPneumProc, PROCEDURE_DATE >= RAD_DATE)
dfPneumProc$ProcDays <- dfPneumProc$PROCEDURE_DATE - dfPneumProc$RAD_DATE

dfPneumProc <- subset(dfPneumProc, dfPneumProc$ProcDays < 365)      #keep procedures
within 365 days of study

setwd('F:\\Inbound\\CTSI\\ELivingston_23_23-001036\\Analysis')
write.csv(dfPneumProc,"PneumProc.csv")


######################################################

##############  GET MORTALITY DATA

setwd('F:\\Inbound\\CTSI\\ELivingston_23_23-001036\\Analysis')

df <- read.csv("F:\\Inbound\\CTSI\\ELivingston_23_23-
001036\\Data\\Patient_Demographics.csv")
#df <- read.csv("F:\\Inbound\\CTSI\\ELivingston_23_23-001036\\Data\\Imaging.csv")
#get CSN information to match with Order ID
dfID <- read.csv("PosPneumPatID.csv")

dfID <- subset(dfID,select=c('IP_PATIENT_ID','RESULT_TIME'))   #Get in-hospital
mortality data in Demographics file
dfID <- dfID %>% rename("RAD_DATE"="RESULT_TIME")
dfID$RAD_DATE <- as.Date(dfID$RAD_DATE,format = "%m/%d/%Y")
dfID <- dfID %>% distinct(IP_PATIENT_ID, .keep_all = TRUE)     #Deduplicate patient
IDs
dfData <- dfID %>%  inner_join(df,by="IP_PATIENT_ID")
dfData <- dfData %>% rename("IN_HOSP_MORT"="VITAL_STATUS")
dfData$IN_HOSP_MORT <- ifelse(dfData$IN_HOSP_MORT=="Known Deceased",1,0)
```

```
df <- read.csv("F:\\Inbound\\CTSI\\ELivingston_23_23-
001036\\Data\\Patient_Identifiers.csv")   #Get date of death data from Pt identifier
file
dfData2 <- dfID %>%  inner_join(df,by="IP_PATIENT_ID")
dfData2$DEATH_DATETIME <- as.Date(dfData2$DEATH_DATETIME,format = "%m/%d/%Y")
dfData2$DeathDays <- dfData2$DEATH_DATETIME - dfData2$RAD_DATE
x <- dfData %>%  inner_join(dfData2,by=c("IP_PATIENT_ID","RAD_DATE"))
write.csv(x,"Patient_Demographics.csv")


##################################################################

##### UPDATE MASTERFILE

dfM <- read.csv("MasterRadPath2.csv")
dfP <- read.csv("PneumProc.csv")
dfGI <- subset(dfP, PROCEDURE_CODE >= "40490" & PROCEDURE_CODE <= "49999") #Keep GI
procedures
dfGI <-
subset(dfGI,select=c('IP_PATIENT_ID','PROCEDURE_DATE','PROCEDURE_CODE','PROCEDURE_DESC
RIPTION','ProcDays'))
dfGI <- dfGI %>% rename("DAYS_RAD_PROC"="ProcDays")
dfALL <- merge(dfM,dfGI,by="IP_PATIENT_ID", all.x = TRUE)  #Merge keeps all rows on
left and right
#dfALL <- subset(dfALL,select = -c(X.x,RAD_DATE.y,X.y))
#dfALL <- dfALLrename("RAD_DATE"="RAD_DATE.x")
dfALL <- subset(dfALL,select = -c(X))
write.csv(dfALL,"MasterPathProc.csv")

#### UPDATE MASTRE FILE WITH MORTALITY DATA

df <- read.csv("MasterPathProc.csv")
df1 <- read.csv("Patient_Demographics.csv")
dfALL <- merge(df,df1,by=c("IP_PATIENT_ID","RAD_DATE"), all.x = TRUE)  #Merge keeps
all rows on left and right
dfALL <- subset(dfALL,select = -c(X.x,X.y,X.1))
write.csv(dfALL,"MasterPathProc.csv")



length(unique(dfPneumProc$IP_PATIENT_ID)) #n=314
length(unique(dfID$IP_PATIENT_ID)) #n=316


############################################################################

############  IDENTIFY ABDOMINAL OPERATIONS

df <- read.csv("MasterPathProc.csv")
df$PROCEDURE_CODE <- as.numeric(df$PROCEDURE_CODE)

resect <- df$PROCEDURE_CODE >= 45.62 & df$PROCEDURE_CODE <= 45.82 |
  df$PROCEDURE_CODE >= 44120 & df$PROCEDURE_CODE <= 44125 |
  df$PROCEDURE_CODE >= 44140 & df$PROCEDURE_CODE <= 44160 |
  df$PROCEDURE_CODE >= 44202 & df$PROCEDURE_CODE <= 44205
df$RESECT <- ifelse(resect,1,0)

anast <- df$PROCEDURE_CODE >= 45.82 & df$PROCEDURE_CODE <= 45.93 |
  df$PROCEDURE_CODE == 44603 |
  df$PROCEDURE_CODE == 44130
df$ANAST <- ifelse(anast,1,0)
```

```
ostomy <- df$PROCEDURE_CODE >= 46.01 & df$PROCEDURE_CODE <= 46.22 |
  df$PROCEDURE_CODE == 44320
df$OSTOMY <- ifelse(ostomy,1,0)

adhesions <- df$PROCEDURE_CODE == 44005 | df$PROCEDURE_CODE == 44180
df$ADHESIONS <- ifelse(adhesions,1,0)

exlap <- df$PROCEDURE_CODE >= 49000 & df$PROCEDURE_CODE <= 49002 |
  df$PROCEDURE_CODE == 49320
df$EXLAP <- ifelse(exlap,1,0)

write.csv(df,"MasterPathProc.csv")

################################################################################
#################  STATISTICS

pacman::p_load(tidyverse,tidytext,data.table,stringi)
setwd("C:\\Users\\ehlsu\\OneDrive\\Research\\Pneumatosis intestinalis\\Old Files")
dfMaster <- read.csv("MasterPathProc.csv")
df <- readxl::read_xlsx("TimeToSurg_Kyle_OpFindings EL CORRECTIONS 110423.xlsx")
length(unique(df$IP_PATIENT_ID))  #n=315 patients with pneumatosis

df$DeathDays <- as.numeric(df$DeathDays)

Death_30_day <- df[(df$DeathDays <=30 & df$DeathDays >=0 & !is.na(df$DeathDays)),]
length(unique(Death_30_day$IP_PATIENT_ID))  #n=71 patients died in house within 30
days

Death_90_day <- df[(df$DeathDays <=90 & df$DeathDays >=0 & !is.na(df$DeathDays)),]
length(unique(Death_90_day$IP_PATIENT_ID))  #n=91 patients died in house within 30
days

df$LapOnly <- as.numeric(df$EXLAP) +  as.numeric(df$ADHESIONS)
df$AllResect <- as.numeric(df$RESECT)+as.numeric(df$OSTOMY)+as.numeric(df$ANAST)

#### TEST CLASSIFICATION (CORRECTED 11/6/23)

dfTEMP <- df
df <- dfTEMP[dfTEMP$AnySurg==1,] #74 patients had surgery

x <- df[df$LapOnly>=1,]
length(unique(x$IP_PATIENT_ID))  #n=23

x <- df[df$EXLAP==1,]
length(unique(x$IP_PATIENT_ID))  #n=15

x <- df[df$ADHESIONS==1,]
length(unique(x$IP_PATIENT_ID))  #n-8

x <- df[df$AllResect>=1,]
length(unique(x$IP_PATIENT_ID))  #n=51

x <- df[df$ANAST>=1,]
length(unique(x$IP_PATIENT_ID))  #n=4

x <- df[df$RESECT>=1,]
length(unique(x$IP_PATIENT_ID))  #n=44

x <- df[df$OSTOMY>=1,]
length(unique(x$IP_PATIENT_ID))  #n=4

y <- df[,c("IP_PATIENT_ID","AllResect","ANAST","RESECT","OSTOMY")]
```

```
y <- y %>% distinct(IP_PATIENT_ID,.keep_all = TRUE)    #Deduplicate

x <- y[y$AllResect>=1,]
length(unique(x$IP_PATIENT_ID))  #n=51


#########################

XLAP_30_day <- df[(df$Days_Rad_Proc <=30 & df$Days_Rad_Proc >=0 & df$LapOnly ==1 &
!is.na(df$DeathDays)),]
length(unique(XLAP_30_day$IP_PATIENT_ID))  #n=15

RESECT_30_day <- df[(df$Days_Rad_Proc <=30 & df$Days_Rad_Proc >=0 & df$RESECT ==1 &
!is.na(df$DeathDays)) ,]
length(unique(RESECT_30_day$IP_PATIENT_ID)) # n=24 deaths for




#####     TABLE 3 SURVIVAL AT 30 AND 90 DAYS (CORRECTED 11/6/23)

df$Day30Mort <- (df$DeathDays <=30 & df$DeathDays >=0 & !is.na(df$DeathDays))
df$Day90Mort <- (df$DeathDays <=90 & df$DeathDays >=0 & !is.na(df$DeathDays))

df$Days_Rad_Proc <- as.numeric(df$Days_Rad_Proc)
df$SurgGT3days <- df$Days_Rad_Proc >= 3 & df$Days_Rad_Proc >=0 &
!is.na(df$Days_Rad_Proc)
df$SurgLE2days <- df$Days_Rad_Proc <= 2 & df$Days_Rad_Proc >=0 &
!is.na(df$Days_Rad_Proc)

sum(df$Day30Mort) #n=17
sum(df$Day90Mort) #n=25
sum(df$Dead90) #n=25
sum(df$SurgGT3days) #12
sum(df$SurgLE2days) #62
temp <- df %>% summarize(DeathDays,Day30Mort,Day90Mort,Dead90)

table(SurgLE2days=df$SurgLE2days,Day30Mort=df$Day30Mort,AnyResect=df$AnyResect)
table(SurgLE2days=df$SurgLE2days,Day90Mort=df$Day90Mort,AnyResect=df$AnyResect)
table(SurgGT3days=df$SurgGT3days,Day30Mort=df$Day30Mort,AnyResect=df$AnyResect)
table(SurgGT3days=df$SurgGT3days,Day90Mort=df$Day90Mort,AnyResect=df$AnyResect)

dfNoSurg <- dfTEMP[dfTEMP$AnySurg==0,]  #n=241 no surgery patients

dfNoSurg$Day30Mort <- (dfNoSurg$DeathDays <=30 & dfNoSurg$DeathDays >=0 &
!is.na(dfNoSurg$DeathDays))
dfNoSurg$Day90Mort <- (dfNoSurg$DeathDays <=90 & dfNoSurg$DeathDays >=0 &
!is.na(dfNoSurg$DeathDays))

sum(dfNoSurg$Day30Mort) #54
sum(dfNoSurg$Day90Mort) #66

#####################################################################################
########


########################################### CREATE DEAD AT 90 DAYS File

df <- read.csv("MasterPathProc.csv")
df1 <- df[df$DeathDays >= 0 & df$DeathDays <= 90,]
df1 <- df1 %>% drop_na("DeathDays")
df1 <- df1 %>% distinct(IP_PATIENT_ID,.keep_all = TRUE)    #Deduplicate
write.csv(df1,"Dead90Days.csv")
```

```
newdf <- df[!(df$IP_PATIENT_ID %in% df1$IP_PATIENT_ID),]   #Keep rows in main database
(df) that are NOT in subset (df1)
newdf <- newdf %>% distinct(IP_PATIENT_ID,.keep_all = TRUE)    #Deduplicate
write.csv(newdf,"NotDead90Days.csv")


##################################################################################

##############  GET ENCOUNTER DIAGNOSES

pacman::p_load(tidyverse,tidytext,data.table,stringi)
setwd('F:\\Inbound\\CTSI\\ELivingston_23_23-001036\\Analysis')
setwd('F:\\Inbound\\CTSI\\ELivingston_23_23-001036\\Data')
df <- read.csv("F:\\Inbound\\CTSI\\ELivingston_23_23-
001036\\Data\\Encounter_Diagnoses.csv")
dfID <- read.csv("PosPneumPatID.csv")

dfID <- subset(dfID,select=c('IP_PATIENT_ID','RESULT_TIME'))   #Get in-hospital
mortality data in Demographics file
dfID <- dfID %>% rename("RAD_DATE"="RESULT_TIME")
dfID$RAD_DATE <- as.Date(dfID$RAD_DATE,format = "%m/%d/%Y")
dfID <- dfID %>% distinct(IP_PATIENT_ID, .keep_all = TRUE)     #Deduplicate patient
IDs

dfData <- dfID %>%  inner_join(df,by="IP_PATIENT_ID")
dfData$DIAGNOSIS_DATE <- as.Date(dfData$DIAGNOSIS_DATE,format = "%m/%d/%Y")
dfData$DAYS_RAD_DX <- dfData$DIAGNOSIS_DATE - dfData$RAD_DATE

setwd('F:\\Inbound\\CTSI\\ELivingston_23_23-001036\\Analysis')
write.csv(dfData,"Patient_Diagnoses.csv")

####################################################################################

#############   CREATE DIAGNOSES FILES FOR DEATHS/NON DEATHS

pacman::p_load(tidyverse,tidytext,data.table,stringi)
setwd("C:\\Users\\ehlsu\\OneDrive\\Research\\Pneumatosis intestinalis")

write.csv(dfData,"Patient_Diagnoses.csv")

pacman::p_load(tidyverse,tidytext,data.table,stringi)
setwd("C:\\Users\\ehlsu\\OneDrive\\Research\\Pneumatosis intestinalis")
dfDx <- read.csv("Patient_Diagnoses.csv")
dfID <- read.csv(("Dead90Days.csv"))   # 99 Patients died by 90 days
dfEnc <- read.csv("PosPneumPatIDEnc.csv")

dfx <- subset(dfEnc,select=c("IP_PATIENT_ID","IP_ORDER_PROC_ID","IP_ENC_ID"))
dfy <- merge(dfID,dfx,by.x=c("IP_PATIENT_ID","RAD_ORDER_ID"),
by.y=c("IP_PATIENT_ID","IP_ORDER_PROC_ID"))   #Merge to get Encounter ID

dfz <- merge(dfy,dfDx, by=c("IP_PATIENT_ID","IP_ENC_ID"))

length(unique(dfz$IP_PATIENT_ID))

write.csv(dfz,"Died90Diagnoses.csv")

###################################################################################

############    ASSIGN CHARLSON/ELIXHAUSER SCORES

# library(githubinstall)
# githubinstall("icd")   # #need RTools to load package - ICD does not work
```

```
pacman::p_load(tidyverse,tidytext,data.table,stringi,touch,comorbidity)
setwd("C:\\Users\\ehlsu\\OneDrive\\Research\\Pneumatosis intestinalis")
df <- read.csv("Died90Diagnoses.csv")  #Read in diagnosis file for patients who died

x <- subset(df,select=c(IP_ENC_ID,ICD_TYPE,ICD_CODE))
x$Convert <- ifelse(df$ICD_TYPE=="9",icd_map(df$ICD_CODE,from = 9, to =
10),df$ICD_CODE <-df$ICD_CODE)

dfc <- comorbidity(x=x, id="IP_ENC_ID", code="Convert",map = "charlson_icd10_quan",
assign0 = FALSE)
dfe <- comorbidity(x=x, id="IP_ENC_ID", code="Convert",map = "elixhauser_icd10_quan",
assign0 = FALSE)


dfc$score <- score(x = dfc, weights = "quan", assign0 = FALSE)
dfe$score <- score(x = dfe, weights = "vw", assign0 = FALSE)




#df$SubsAbuse <- ifelse(between(df$ICD_CODE,"F10","F15"),1,0) #ETOH and Substance
Abuse

write.csv(df,"Temp.csv")

# https://cran.r-project.org/web/packages/comorbidity/comorbidity.pdf

##################################################################################

##### RISK RACTORS AND MORTALITY (ELIXHAUSER/CHARLSON)



pacman::p_load(tidyverse,tidytext,data.table,stringi,touch,comorbidity)
setwd("C:\\Users\\ehlsu\\OneDrive\\Research\\Pneumatosis intestinalis")#dfEnc <-
read.csv("PosPneumPatIDEnc.csv")  #Read file with IDs
dfMast <- read.csv("MasterPathProc.csv")  #Read master file
dfID <- subset(dfEnc,select=c("IP_PATIENT_ID","IP_ORDER_PROC_ID","IP_ENC_ID")) #Get
encounter ID
#dfy <- merge(dfMast,dfID,by.x=c("IP_PATIENT_ID","RAD_ORDER_ID"),
by.y=c("IP_PATIENT_ID","IP_ORDER_PROC_ID"))   #add encounter ID to Master
#write.csv(dfy,"MasterPathProc.csv")

dfDx <- read.csv("Patient_Diagnoses.csv")  #read dx file
dfMastDx <- merge(dfMast,dfDx, by=c("IP_PATIENT_ID","IP_ENC_ID")) #add dx to master
file

x <- "(\"PRIMARY_DIAGNOSIS_FLAG\" == \"P\") | (\"PRESENT_ON_ADMISSION\" == 1)"
dfx <- dfMastDx[(dfMastDx$PRIMARY_DIAGNOSIS_FLAG == "P") |
(dfMastDx$PRESENT_ON_ADMISSION == 1),] #Keep POA and primary dx
length(unique(dfx$IP_PATIENT_ID))   # 277 of the 316 patients have POA and primary dx
flags
dfx$Dead90 <- ifelse((dfx$DeathDays >= 0 & dfx$DeathDays <= 90),1,0)  #92 unique
deaths

#### GET ELIX/CHAR CODES

dfx$Convert <- ifelse(dfx$ICD_TYPE=="9",icd_map(dfx$ICD_CODE,from = 9, to =
10),dfx$ICD_CODE <-dfx$ICD_CODE)
```

```
dfc <- comorbidity(x=dfx, id="IP_ENC_ID", code="Convert",map = "charlson_icd10_quan", 
assign0 = FALSE)
dfe <- comorbidity(x=dfx, id="IP_ENC_ID", code="Convert",map = 
"elixhauser_icd10_quan", assign0 = FALSE)


dfc$score <- score(x = dfc, weights = "quan", assign0 = FALSE)
dfe$score <- score(x = dfe, weights = "vw", assign0 = FALSE)

dfc <- merge(dfc,dfID, by="IP_ENC_ID") #add ID
dfe <- merge(dfe,dfID, by="IP_ENC_ID") #add ID

x <- subset(dfx,select=c("IP_ENC_ID","Dead90"))  #Add dead at 90 days
x <-  x[!duplicated(x[c('IP_ENC_ID')]),]   #deduplicate Encounters

dfc <- merge(x=dfc,y=x,by="IP_ENC_ID")
dfe <- merge(x=dfe,y=x,by="IP_ENC_ID")   #277 encounters

dfc["Dead90"][is.na(dfc["Dead90"])] <- 0  #replace NA in column Dead990 with 0
dfe["Dead90"][is.na(dfe["Dead90"])] <- 0  #329 encounters for 277 patients

dfc <-  dfc[!duplicated(dfc[c('IP_ENC_ID')]),]   #deduplicate Encounters
dfe <-  dfe[!duplicated(dfe[c('IP_ENC_ID')]),]

dfDate <- subset(dfMast,select=c("RAD_DATE","IP_ENC_ID")) #Get Rad date per encounter#
dfDate <-  dfDate[!duplicated(dfDate[c('IP_ENC_ID')]),]   #deduplicate Encounters in 
Date
dfc <- merge(x=dfc,y=dfDate,by="IP_ENC_ID")
dfe <- merge(x=dfe,y=dfDate,by="IP_ENC_ID") # Add Rad date to Elix/Char files




write.csv(dfc,"CharWork.csv")  #Write comorbidity working files
write.csv(dfe,"ElixWork.csv")


temp <- dfx[dfx$Dead90==1,]
length(unique(dfe$IP_PATIENT_ID))
length(unique(dfe$IP_ENC_ID))
length(unique(temp$IP_PATIENT_ID))

##### TABLE OF ELIX/CHAR RESULTS


library(stddiff)
library(cobalt)
library(table1)
library(Hmisc)

pacman::R_load(stddiff,cobalt,table1,Hmisc)

# SMD FUNCTION----------------------------------------------------------------
SMD_value <- function(x, ...) {
  # Construct vectors of data y, and groups (strata) g
  y <- unlist(x)
  g <- factor(rep(1:length(x), times=sapply(x, length)))
  if (is.numeric(y)) {
    # For numeric variables
    try({a<-data.frame(y)
    a$g<-g
    smd<-(as.data.frame(stddiff.numeric(data=a,gcol = "g", vcol = "y")))$stddiff
    },silent=TRUE)
```

```
  } else {
    # For categorical variables
    try({
      a<-data.frame(y)
      a$g<-g
      smd<-(abs((bal.tab(a, treat = "g",data=a,binary="std",continuous =
                          "std",s.d.denom =
"pooled",stats=c("mean.diffs"))$Balance)$Diff.Un))
    },silent=TRUE)
  }
  c("",format(smd,digits=2)) #Formatting number of digits
}

# CONTINUOUS VARIABLES FORMATTING-----------------------------------------------
my.render.cont <- function(x) {
  with(stats.default(x),
       c("",

         "Mean (SD)" = sprintf("%s (%s)",
                              round_pad(MEAN, 1),
                              round_pad(SD, 1)),

         "Median (IQR)" = sprintf("%s (%s, %s)",
                                 round_pad(MEDIAN, 1),
                                 round_pad(Q1, 1),
                                 round_pad(Q3, 1)))
  )}

###### Creating the final table----------------------------------------------------


dfe <- read.csv("ElixWork.csv")

dfe$Dead90 <- factor(dfe$Dead90, levels=c(0,1),
                     labels=c("Alive",
                              "Dead"))

dfe$chf <- factor(dfe$chf, levels=c(0,1),
                    labels=c("No CHF",
                             "CHF"))

dfe$carit <- factor(dfe$carit, levels=c(0,1),
                  labels=c("No ARRYTHM",
                           "ARRYTHMIA"))

dfe$valv <- factor(dfe$valv, levels=c(0,1),
                    labels=c("No VALVULAR DISEASE",
                             "VALVULAR DISEASE"))

dfe$pcd <- factor(dfe$pcd, levels=c(0,1),
                  labels=c("",
                           "PUMONARY CIRCULATION DISORDERS"))

dfe$pvd <- factor(dfe$pvd, levels=c(0,1),
                  labels=c("",
                           "PERIPHERAL VASCULAR DISEASE"))

dfe$hypunc <- factor(dfe$hypunc, levels=c(0,1),
                  labels=c("",
                           "UNCOMPLICATED HTN"))

dfe$hypc <- factor(dfe$hypc, levels=c(0,1),
                     labels=c("",
```

```
                              "COMPLICATED HTN"))

dfe$para <- factor(dfe$para, levels=c(0,1),
                   labels=c("",
                            "PARALYSIS"))

dfe$ond <- factor(dfe$ond, levels=c(0,1),
                  labels=c("",
                           "OTHER NEUROLOGIC DISORDERS"))
dfe$cpd <- factor(dfe$cpd, levels=c(0,1),
                  labels=c("",
                           " CHRONIC PULMONARY DISEASE "))

dfe$diabunc <- factor(dfe$diabunc, levels=c(0,1),
                      labels=c("",
                               "UNCOMPLICATED DIABETES "))

dfe$diabc <- factor(dfe$diabc, levels=c(0,1),
                    labels=c("", "COMPLICATED DIABETES "))

dfe$hypothy <- factor(dfe$hypothy, levels=c(0,1),
                      labels=c("", " HYPOTHYROIDISM"))

dfe$rf <- factor(dfe$rf, levels=c(0,1),
                 labels=c("", " RENAL FAILURE"))

dfe$ld <- factor(dfe$ld, levels=c(0,1),
                 labels=c("", "LIVER DISEASE"))

dfe$pud <- factor(dfe$pud, levels=c(0,1),
                  labels=c("", "PEPTIC ULCER DISEASE, EXCLUDING BLEEDING "))

dfe$aids <- factor(dfe$aids, levels=c(0,1),
                   labels=c("", "AIDS/HIV "))

dfe$lymph <- factor(dfe$lymph, levels=c(0,1),
                    labels=c("", "LYMPHOMA"))

dfe$metacanc <- factor(dfe$metacanc, levels=c(0,1),
                    labels=c("", "METASTATIC CANCER"))

dfe$solidtum <- factor(dfe$solidtum, levels=c(0,1),
                       labels=c("", " SOLID TUMOR, WITHOUT METASTASIS "))

dfe$rheumd <- factor(dfe$rheumd, levels=c(0,1),
                     labels=c("", "RHEUMATOID ARTHRITIS/COLLAGED VASCULAR DISEASE"))

dfe$coag <- factor(dfe$coag, levels=c(0,1),
                   labels=c("", "COAGULOPATHY"))

dfe$obes <- factor(dfe$obes, levels=c(0,1),
                   labels=c("", "OBESITY"))

dfe$wloss <- factor(dfe$wloss, levels=c(0,1),
                    labels=c("", "WEIGHT LOSS "))

dfe$fed <- factor(dfe$fed, levels=c(0,1),
                  labels=c("", "FLUID AND ELECTROLYTE DISORDERS"))

dfe$blane <- factor(dfe$blane, levels=c(0,1),
                    labels=c("", "BLOOD LOSS ANAEMIA "))

dfe$dane <- factor(dfe$dane, levels=c(0,1),
```

```
                   labels=c("", "DEFICIENCY ANAEMIA"))

dfe$alcohol <- factor(dfe$alcohol, levels=c(0,1),
                      labels=c("", "ALCOHOL ABUSE"))

dfe$drug <- factor(dfe$drug, levels=c(0,1),
                   labels=c("", "DRUG ABUSE"))

dfe$psycho <- factor(dfe$psycho, levels=c(0,1),
                     labels=c("", "PSYCHOSES"))

dfe$depre <- factor(dfe$depre, levels=c(0,1),
                    labels=c("", "DEPRESSION"))



#label(dfe$chf) <- "CHF"
#label(dfe$carit) <- "CARDIAC ARRYTHMIA"
#label(dfe$valv) <- "VALVULAR DISEASE"

Table1<-table1(~ chf + carit + valv + pcd + pvd + hypunc +
                 hypc + para + ond + cpd + diabunc + diabc +
                 hypothy + rf + ld + pud +  aids + lymph +metacanc + solidtum +
                 rheumd + coag + obes + wloss + fed + blane + dane +
                 alcohol + drug + psycho + depre + score | Dead90,
               data=dfe,
               overall = FALSE,
               render.continuous = my.render.cont,
               extra.col=list(`SMD`=SMD_value))

Table1

###################### ALTERNATIVE TABLE-CAN USE TO CHECK SMD CALCS

library(tableone)
dfe <- read.csv("ElixWork.csv")
vars <- c("chf","carit","valv","score")
dfe$chf <- factor(dfe$chf, levels=c(0,1),
                  labels=c("No CHF",
                           "CHF"))
#label(dfe$chf) <- "CHF"
tabUnmatched <- CreateTableOne(vars = vars, strata = "Dead90", data = dfe, test =
FALSE)
print(tabUnmatched, smd = TRUE)

######################

# https://cran.r-project.org/web/packages/table1/vignettes/table1-examples.html
# https://cran.r-project.org/web/packages/tableone/vignettes/smd.html
# https://cran.r-project.org/web/packages/smd/vignettes/smd_usage.html
# https://cran.r-project.org/web/packages/comorbidity/comorbidity.pdf

#################################################################################

###################### ADD INDICATORS FOR DEATH AT 90 DAYS AND 1ST PNEUM ENCOUNTER TO
MASTER FILE

df <- read.csv("MasterPathProc.csv")
df1 <- df[df$DeathDays >= 0 & df$DeathDays <= 90,]
df1 <- df1 %>% drop_na("DeathDays")
df1 <- df1 %>% distinct(IP_PATIENT_ID,.keep_all = TRUE)    #Deduplicate
write.csv(df1,"Dead90Days.csv")
```

```
################################################################################

############### ADD DUMMY TO MASTER FOR DEAD AT 90 DAYS AND FIRST PNEUM DX

pacman::p_load(tidyverse,tidytext,data.table,stringi,touch,comorbidity)
df <- read.csv("MasterPathProc.csv")

dfTemp <- df %>%                                  #Keep score for date of 1st pneum
rad date
  group_by(IP_PATIENT_ID) %>%
  slice_min(as.Date(RAD_DATE),with_ties = FALSE) %>%
  ungroup()

write.csv(dfTemp,"MasterPathProcFirstPneum")

dfTemp <- dfTemp %>% distinct(IP_PATIENT_ID,.keep_all = TRUE)    #Deduplicate
df$FirstPneum =(as.integer(df$IP_ENC_ID %in% dfTemp$IP_ENC_ID))

length(unique(df$IP_PATIENT_ID)) #n=316
df1 <- df[df$FirstPneum==1,]
length(unique(df1$IP_PATIENT_ID)) #n=316, every patient has a dummy for the 1st pneum
encounter

df$Dead90 <- ifelse((df$DeathDays >= 0 & df$DeathDays <= 90),1,0)
df["Dead90"][is.na(df["Dead90"])] <- 0  #rep


df1 <- df[df$Dead90==1,]
length(unique(df1$IP_PATIENT_ID)) #n=99 patients who died

write.csv(df,"MasterPathProc1.csv")

###############################################################################

##### CREATE FILES FOR EACH CATEGORY OF ALIVE/DEAD, LAP ONLY/BOWEL RESECTION

pacman::p_load(tidyverse,tidytext,data.table,stringi,touch,comorbidity)
df <- read.csv("MasterPathProc1.csv")

dfDead <- df[df$Dead90==1,]
length(unique(dfDead$IP_PATIENT_ID))   #99 dead pts
dfDeadLap <- dfDead[dfDead$LapOnly==1,]
length(unique(dfDeadLap$IP_PATIENT_ID))   #9 dead, neg lap
dfDeadResect <- dfDead[dfDead$AllResect==1,]
length(unique(dfDeadResect$IP_PATIENT_ID))   #22 dead, neg lap

write.csv(dfDeadLap,"DeadLapn9.csv")
write.csv(dfDeadResect,"DeadResectn22.csv")

dfAlive <- df[df$Dead90==0,]
length(unique(dfAlive$IP_PATIENT_ID))   #217 Alive pts

dfAliveLap <- dfAlive[dfAlive$LapOnly==1,]
length(unique(dfAliveLap$IP_PATIENT_ID))   #20 Alive, neg lap
dfAliveResect <- dfAlive[dfAlive$AllResect==1,]
length(unique(dfAliveResect$IP_PATIENT_ID))   #30 Alive, neg lap

write.csv(dfAliveLap,"AliveLapn20.csv")
write.csv(dfAliveResect,"AliveResectn30.csv")

###################### FIX Dead90 for paients with multiple records

pacman::p_load(tidyverse,tidytext,data.table,stringi,touch,comorbidity)
```

```
df <- read.csv("MasterPathProc1.csv")
df$LapOnly <- df$EXLAP +  df$ADHESIONS
df$AllResect <- df$RESECT+df$OSTOMY+df$ANAST
df["LapOnly"][is.na(df["LapOnly"])] <- 0  #
df["AllResect"][is.na(df["AllResect"])] <- 0  #

length(unique(df$IP_PATIENT_ID)) #n=316 patients
df1 <- df[df$Dead90==1,]
length(unique(df1$IP_PATIENT_ID))  #n=99 died
df1 <- df[df$FirstPneum==1,]
length(unique(df1$IP_PATIENT_ID))  #n=316 have FirstPneum assigned
df1 <- df[df$LapOnly==1,]
length(unique(df1$IP_PATIENT_ID))  #n=30 have FirstPneum assigned
df1 <- df[df$AllResect==1,]
length(unique(df1$IP_PATIENT_ID))  #n=53 have FirstPneum assigned


dfDead <- subset(df,select=c("IP_PATIENT_ID","Dead90"))
dfDead <- dfDead[dfDead$Dead90==1,]
dfDead1 <- dfDead %>% distinct(IP_PATIENT_ID,.keep_all = TRUE)  #Pt ID for the 99
deaths
df$Dead90 <- (as.integer((df$IP_PATIENT_ID %in% dfDead1$IP_PATIENT_ID)))

dfDead <- subset(df,select=c("IP_PATIENT_ID","LapOnly"))
dfDead <- dfDead[dfDead$LapOnly==1,]
dfDead1 <- dfDead %>% distinct(IP_PATIENT_ID,.keep_all = TRUE)  #Pt ID for the 99
deaths
df$LapOnly <- (as.integer((df$IP_PATIENT_ID %in% dfDead1$IP_PATIENT_ID)))

dfDead <- subset(df,select=c("IP_PATIENT_ID","AllResect"))
dfDead <- dfDead[dfDead$AllResect==1,]
dfDead1 <- dfDead %>% distinct(IP_PATIENT_ID,.keep_all = TRUE)  #Pt ID for the 99
deaths
df$AllResect <- (as.integer((df$IP_PATIENT_ID %in% dfDead1$IP_PATIENT_ID)))

length(unique(df$IP_PATIENT_ID)) #n=316 patients
df1 <- df[df$Dead90==1,]
length(unique(df1$IP_PATIENT_ID))  #n=99 died
df1 <- df[df$FirstPneum==1,]
length(unique(df1$IP_PATIENT_ID))  #n=316 have FirstPneum assigned
df1 <- df[df$LapOnly==1,]
length(unique(df1$IP_PATIENT_ID))  #n=29 have FirstPneum assigned
df1 <- df[df$AllResect==1,]
length(unique(df1$IP_PATIENT_ID))  #n=52 have FirstPneum assigned

write.csv(df,"MasterPathProc1.csv")

#####  ANALYTIC TABLES

df <- read.csv("MasterPathProc1.csv")
df1 <- df %>% distinct(IP_PATIENT_ID,.keep_all = TRUE)  #Pt

length(unique(df1$IP_PATIENT_ID)) #n=316 patients
dfTemp <- df1[df1$Dead90==1,]
length(unique(dfTemp$IP_PATIENT_ID)) #99 deaths

table(df1$LapOnly,df1$Dead90)
table(df1$AllResect,df1$Dead90)

#################################################################################

########## GET PROVIDER NOTES
```

```
pacman::p_load(tidyverse,tidytext,data.table,stringi,touch,comorbidity,data.table)
pacman::p_load(RSQLite)
con <- dbConnect("SQLite", dbname = "sample_db.sqlite")



dbWriteTable(con, name="sample_table", value="Provider_Notes.csv",
    row.names=FALSE, header=TRUE, sep = ",")

# Query your data as you like
yourData <- dbGetQuery(con, "SELECT * FROM sample_table LIMIT 10")

dbDisconnect(con)

setwd('F:\\Inbound\\CTSI\\ELivingston_23_23-001036\\Analysis')
dfID <- read.csv("DeadLapn9.csv")
setwd('F:\\Inbound\\CTSI\\ELivingston_23_23-001036\\Data')
dfPN <- fread("Provider_Notes.csv")

dfID <-  dfID[!duplicated(dfID[,c('IP_PATIENT_ID')]),]
dfID <- subset(dfID,"IP_PATIENT_ID")
dfTemp <- left_join(x=dfPN,y=dfID,by = "IP_PATIENT_ID")   #add

setwd('F:\\Inbound\\CTSI\\ELivingston_23_23-001036\\Analysis')
write.csv(dfTemp,"DeapLapNotes.csv")


##################################################################################

################################  GET PNEUM DATE

dfRad <- read.csv("PosPneumPatID.csv")
dfPath <- read.csv("PneumPathResults.csv")

dfRad$RESULT_TIME <- as.Date(dfRad$RESULT_TIME,format = "%m/%d/%Y")   #Convert to date
formsat
dfPath$ORDER_TIME <- as.Date(dfPath$ORDER_TIME,format = "%m/%d/%Y")

dfRadFirstDate <- dfRad %>% group_by(IP_PATIENT_ID) %>%                #Cut data for
first radiology date for any patient only (n=316 patients)
  slice(which.min(RESULT_TIME))

dfRadFirstDate  <- subset(dfRadFirstDate, select=c(IP_PATIENT_ID,RESULT_TIME))  #keep
only patient ID and first radiology image date
dfRadFirstDate <- dfRadFirstDate %>% rename("PNEUM_TIME"="RESULT_TIME")
write.csv(dfRadFirstDate,"RadFirstDate.csv")

##################################################################################

######################  LABS

### DeadLap

pacman::p_load(tidyverse,tidytext,data.table,stringi,data.table)

setwd('F:\\Inbound\\CTSI\\ELivingston_23_23-001036\\Data')
dfLab <- fread("Labs.csv")
setwd('F:\\Inbound\\CTSI\\ELivingston_23_23-001036\\Analysis')
dfID <- read.csv("DeadLapn9.csv")

dfID <-  dfID[!duplicated(dfID[,c('IP_PATIENT_ID')]),] #Only use ID numbers for the
patients in subgroups
dfTemp <- semi_join(x=dfLab,y=dfID,by = "IP_PATIENT_ID")
```

```
length(unique(dfTemp$IP_PATIENT_ID))  #n=9 uniique patients
write.csv(dfTemp,"LabTempn9.csv")   #### TEMP CSV FILE

dfPneumDate <- read.csv("RadFirstDate.csv")
dfTemp1 <- merge(x=dfTemp,y=dfPneumDate,by = "IP_PATIENT_ID")
dfTemp1 <- dfTemp1[,-("X")]
dfTemp1$ORDER_TIME <- as.Date(dfTemp1$ORDER_TIME,format = "%m/%d/%Y")
dfTemp1$PNEUM_TIME <- as.Date(dfTemp1$PNEUM_TIME)
dfTemp1$TIME_PNEUM_LAB <- dfTemp1$ORDER_TIME  - dfTemp1$PNEUM_TIME

write.csv(dfTemp1,"DeadLapLabn9.csv")   #### FINAL CSV FILE


### DeadResect


dfID <- read.csv("DeadResectn22.csv")

dfID <-  dfID[!duplicated(dfID[,c('IP_PATIENT_ID')]),] #Only use ID numbers for the
patients in subgroups
dfTemp <- semi_join(x=dfLab,y=dfID,by = "IP_PATIENT_ID")
length(unique(dfTemp$IP_PATIENT_ID))  #n=9 uniique patients
write.csv(dfTemp,"LabTempn22.csv")   #### TEMP CSV FILE

dfPneumDate <- read.csv("RadFirstDate.csv")
dfTemp1 <- merge(x=dfTemp,y=dfPneumDate,by = "IP_PATIENT_ID")
dfTemp1 <- dfTemp1[,-("X")]
dfTemp1$ORDER_TIME <- as.Date(dfTemp1$ORDER_TIME,format = "%m/%d/%Y")
dfTemp1$PNEUM_TIME <- as.Date(dfTemp1$PNEUM_TIME)
dfTemp1$TIME_PNEUM_LAB <- dfTemp1$ORDER_TIME  - dfTemp1$PNEUM_TIME

write.csv(dfTemp1,"DeadResectn22.csv")   #### FINAL CSV FILE

### AliveLapn20


dfID <- read.csv("AliveLapn20.csv")

dfID <-  dfID[!duplicated(dfID[,c('IP_PATIENT_ID')]),] #Only use ID numbers for the
patients in subgroups
dfTemp <- semi_join(x=dfLab,y=dfID,by = "IP_PATIENT_ID")
length(unique(dfTemp$IP_PATIENT_ID))  #n=9 uniique patients
#write.csv(dfTemp,"LabTempn22.csv")   #### TEMP CSV FILE

dfPneumDate <- read.csv("RadFirstDate.csv")
dfTemp1 <- merge(x=dfTemp,y=dfPneumDate,by = "IP_PATIENT_ID")
dfTemp1 <- dfTemp1[,-("X")]
dfTemp1$ORDER_TIME <- as.Date(dfTemp1$ORDER_TIME,format = "%m/%d/%Y")
dfTemp1$PNEUM_TIME <- as.Date(dfTemp1$PNEUM_TIME)
dfTemp1$TIME_PNEUM_LAB <- dfTemp1$ORDER_TIME  - dfTemp1$PNEUM_TIME

write.csv(dfTemp1,"AliveLapLabn20.csv")   #### FINAL CSV FILE

### AliveResectn30


dfID <- read.csv("AliveResectn30.csv")

dfID <-  dfID[!duplicated(dfID[,c('IP_PATIENT_ID')]),] #Only use ID numbers for the
patients in subgroups
dfTemp <- semi_join(x=dfLab,y=dfID,by = "IP_PATIENT_ID")
length(unique(dfTemp$IP_PATIENT_ID))  #n=9 uniique patients
#write.csv(dfTemp,"LabTempn22.csv")   #### TEMP CSV FILE
```

```
dfPneumDate <- read.csv("RadFirstDate.csv")
dfTemp1 <- merge(x=dfTemp,y=dfPneumDate,by = "IP_PATIENT_ID")
dfTemp1 <- dfTemp1[,-("X")]
dfTemp1$ORDER_TIME <- as.Date(dfTemp1$ORDER_TIME,format = "%m/%d/%Y")
dfTemp1$PNEUM_TIME <- as.Date(dfTemp1$PNEUM_TIME)
dfTemp1$TIME_PNEUM_LAB <- dfTemp1$ORDER_TIME  - dfTemp1$PNEUM_TIME

write.csv(dfTemp1,"AliveResectLabn30.csv")   #### FINAL CSV FILE

###########################################################################

###################################### CREATE SQL DATABASE

pacman::p_load(tidyverse,tidytext,data.table,stringi,dplyr)

library(tidyverse)
library(DBI)
library(RSQLite)
library(vroom)

setwd('F:\\Inbound\\CTSI\\ELivingston_23_23-001036\\Analysis')
setwd('F:\\Inbound\\CTSI\\ELivingston_23_23-001036\\Data')

mydb <- dbConnect(SQLite(),"Provider_Notes.sqlite3")

read_csv_chunked("Provider_Notes.csv",
                 callback = function(chunk, dummy){
                   dbWriteTable(mydb, "ProviderNotes", chunk, append = T)},
                 chunk_size = 50000,col_types = "ccccccdcc")

#https://www.michaelc-m.com/manual_posts/2022-01-27-big-CSV-SQL.html


dbDisconnect(mydb)

######################################################################

############################## IMPORT NOTES

# Need to set up Provider_Notes as sqlite object as above

##### DeadLap


setwd('F:\\Inbound\\CTSI\\ELivingston_23_23-001036\\Analysis')
dfID <- read.csv("DeadLapLabn9.csv")

setwd('F:\\Inbound\\CTSI\\ELivingston_23_23-001036\\Data')

dfID <-  dfID[!duplicated(dfID[,c('IP_PATIENT_ID')]),] #Only use ID numbers for the
patients in subgroups
dfID <- subset(dfID,select=c("IP_PATIENT_ID","PNEUM_TIME"))

dfPN <- tbl(mydb,"ProviderNotes")

dfTemp <- right_join(x=dfPN,y=dfID,by = "IP_PATIENT_ID",copy=TRUE)

length(unique(dfTemp$IP_PATIENT_ID))  #n=9 uniique patients

######### DeadResect

## Placing the ID files in an SQL format did not help anything
```

```
setwd('F:\\Inbound\\CTSI\\ELivingston_23_23-001036\\Analysis')

TempOp <- dbConnect(SQLite(),"TempOp.sqlite3")

dfID <- read.csv("DeadResectn22.csv")
dfID <-  dfID[!duplicated(dfID[,c('IP_PATIENT_ID')]),] #Only use ID numbers for the
patients in subgroups
dfID <- subset(dfID,select=c("IP_PATIENT_ID","PNEUM_TIME"))
dbWriteTable(TempOp,"dfID",dfID)

dfPN <- tbl(mydb,"ProviderNotes")
dfID2 <- tbl(TempOp,"dfID")

dfTemp <- right_join(x=dfPN,y=dfID2,by = "IP_PATIENT_ID",copy=TRUE)

length(unique(dfTemp$IP_PATIENT_ID))

write.csv(dfTemp,"NOTESDeadLapn22.csv")

############################# AliveLap/AliveResect

setwd('F:\\Inbound\\CTSI\\ELivingston_23_23-001036\\Analysis')
#dfID <- read.csv("AliveLapLabn20.csv")
dfID <- read.csv("AliveResectLabn30.csv")

setwd('F:\\Inbound\\CTSI\\ELivingston_23_23-001036\\Data')

dfID <-  dfID[!duplicated(dfID[,c('IP_PATIENT_ID')]),] #Only use ID numbers for the
patients in subgroups
dfID <- subset(dfID,select=c("IP_PATIENT_ID","PNEUM_TIME"))
#length(unique(dfID$IP_PATIENT_ID))  #n=20 unique AliveLap patients
length(unique(dfID$IP_PATIENT_ID))  #n= 30 unique AliveResect patients

dfPN <- tbl(mydb,"ProviderNotes")

dfTemp <- right_join(x=dfPN,y=dfID,by = "IP_PATIENT_ID",copy=TRUE)

#write.csv(dfTemp,"NOTESAliveLapn20.csv")
write.csv(dfTemp,"NOTESResectLapn30.csv")

########################################################################

####################  MAKE TABLE 2


pacman::p_load(tidyverse,tidytext,data.table,stringi,touch,comorbidity)
df <- read.csv("MasterPathProcFirstPneum.csv")
length(unique(df$IP_PATIENT_ID))  #n=316 have FirstPneum assigned

df$LapOnly <- df$EXLAP +  df$ADHESIONS
df$AllResect <- df$RESECT+df$OSTOMY+df$ANAST
df["LapOnly"][is.na(df["LapOnly"])] <- 0  #
df["AllResect"][is.na(df["AllResect"])] <- 0  #
df$Dead90 <- ifelse((df$DeathDays >= 0 & df$DeathDays <= 90),1,0)
df["Dead90"][is.na(df["Dead90"])] <- 0  #rep

df1 <-
subset(df,select=c("IP_PATIENT_ID","Days_Rad_Proc","AGE","SEX","RACE","ETHNICITY","ADI
_NATIONAL_RANK","ADI_STATE_RANK","DeathDays","Dead90","LapOnly","AllResect"))

write.csv(df1,"Table2.csv")
```

```
######################################################################

############  GET TOP 5 ELIX CONDITIONS AND ADD TO TABLE 2

pacman::p_load(tidyverse,tidytext,data.table,stringi,touch,comorbidity)
setwd("C:\\Users\\ehlsu\\OneDrive\\Research\\Pneumatosis intestinalis")
dfElix<- read.csv("ElixWork.csv")

dfElix <- subset(dfElix,select=c("IP_PATIENT_ID","alcohol","ld","chf","wloss","coag"))
dfElix <- dfElix %>% rename("ETOH"="alcohol")
dfElix <- dfElix %>% rename("LIVER DISEASE"="ld")
dfElix <- dfElix %>% rename("CHF"="chf")
dfElix <- dfElix %>% rename("WEIGHT LOSS"="wloss")
dfElix <- dfElix %>% rename("COAGULOPATHY"="coag")

dfTbl2<- read.csv("Table2.csv")
dfTemp <- left_join(dfTbl2,dfElix,by="IP_PATIENT_ID")

write.csv(dfTemp,"Table2v2.csv")

########################################################################

##########  GET LABS FOR TABLE 2

pacman::p_load(tidyverse,tidytext,data.table,stringi,touch,comorbidity)
setwd('F:\\Inbound\\CTSI\\ELivingston_23_23-001036\\Data')
dfLab <- fread("Labs.csv")

dfLab <- fread("Labs.csv")


dfID <- read.csv("PosPneumPatIDEnc.csv")
dfID <-  dfID[!duplicated(dfID[,c('IP_PATIENT_ID')]),] #Only use ID numbers for the
patients in subgroups
length(unique(dfID$IP_PATIENT_ID)) #316 patients

dfTemp <- semi_join(x=dfLab,y=dfID,by = "IP_PATIENT_ID")
length(unique(dfTemp$IP_PATIENT_ID))  #n=316 uniique patients

write.csv(dfTemp,"LabTempn316.csv")   #### TEMP CSV FILE

setwd('F:\\Inbound\\CTSI\\ELivingston_23_23-001036\\Analysis')
dfPneumDate <- read.csv("RadFirstDate.csv")
dfTemp1 <- merge(x=dfTemp,y=dfPneumDate,by = "IP_PATIENT_ID")
dfTemp1 <- dfTemp1[,-("X")]
dfTemp1$ORDER_TIME <- as.Date(dfTemp1$ORDER_TIME,format = "%m/%d/%Y")
dfTemp1$PNEUM_TIME <- as.Date(dfTemp1$PNEUM_TIME)
dfTemp1$TIME_PNEUM_LAB <- dfTemp1$ORDER_TIME  - dfTemp1$PNEUM_TIME
length(unique(dfTemp1$IP_PATIENT_ID))  #n316 uniique patient

write.csv(dfTemp1,"Labs316.csv")   #### FINAL CSV FILE


###################################################################################


############## SELECT LABS FOR TABLE 2

pacman::p_load(tidyverse,tidytext,data.table,stringi,touch,comorbidity)
setwd("C:\\Users\\ehlsu\\OneDrive\\Research\\Pneumatosis intestinalis")
dfLab316<- read.csv("Labs316.csv")
dfTble2 <- read.csv("Table2v2.csv")
```

```
length(unique(dfLab316$IP_PATIENT_ID))  #n=316
length(unique(dfTble2$IP_PATIENT_ID))  #n=316

dfLab2day <- dfLab316[(dfLab316$TIME_PNEUM_LAB >= -2 & dfLab316$TIME_PNEUM_LAB <= 0),]
write.csv(dfLab2day,"Lab2day.csv")
length(unique(dfLab2day$IP_PATIENT_ID))  #n=293

dfTemp <- dfLab2day[dfLab2day$COMPONENT_NAME == 'WHITE BLOOD CELL COUNT',] #MAX VALUE
length(unique(dfTemp$IP_PATIENT_ID))  #n=290
length(unique(dfLab2day$IP_PATIENT_ID))  #n=293
dfTemp1 <- dfTemp %>%
  group_by(IP_PATIENT_ID) %>%
  summarise(max = max(RESULT, na.rm=TRUE))
length(unique(dfTemp$IP_PATIENT_ID))  #n=290
dfTble2 <- left_join(dfTble2,dfTemp1,by='IP_PATIENT_ID')
dfTble2 <- dfTble2  %>% rename("WBC" = "max")
ungroup(dfTemp)
write.csv(dfTble2,"Table2v3.csv")

dfTemp <- dfLab2day[(dfLab2day$COMPONENT_NAME == 'LACTATE' | dfLab2day$COMPONENT_NAME
== 'BLOOD LACTATE') ,]
length(unique(dfTemp$IP_PATIENT_ID))  #n=290
length(unique(dfLab2day$IP_PATIENT_ID))  #n=293
dfTemp1 <- dfTemp %>%
  group_by(IP_PATIENT_ID) %>%
  summarise(max = max(RESULT, na.rm=TRUE))
length(unique(dfTemp$IP_PATIENT_ID))  #n=290
dfTble2 <- left_join(dfTble2,dfTemp1,by='IP_PATIENT_ID')
dfTble2 <- dfTble2  %>% rename("LACTATE" = "max")
#ungroup(dfTemp)
write.csv(dfTble2,"Table2v3.csv")

dfTemp <- dfLab2day[(dfLab2day$COMPONENT_NAME == 'CK, TOTAL,PLASMA' |
                       dfLab2day$COMPONENT_NAME == 'CREATINE PHOSPHOKINASE, TOTAL'|
                      dfLab2day$COMPONENT_NAME == 'CK, TOTAL') ,]
length(unique(dfTemp$IP_PATIENT_ID))  #n=290
length(unique(dfLab2day$IP_PATIENT_ID))  #n=293
dfTemp1 <- dfTemp %>%
  group_by(IP_PATIENT_ID) %>%
  summarise(max = max(RESULT, na.rm=TRUE))
length(unique(dfTemp$IP_PATIENT_ID))  #n=290
dfTble2 <- left_join(dfTble2,dfTemp1,by='IP_PATIENT_ID')
dfTble2 <- dfTble2  %>% rename("CK" = "max")
#ungroup(dfTemp)
write.csv(dfTble2,"Table2v3.csv")

dfTemp <- dfLab2day[dfLab2day$COMPONENT_NAME == 'TROPONIN INTERPRETATION',]
length(unique(dfTemp$IP_PATIENT_ID))  #n=290
length(unique(dfLab2day$IP_PATIENT_ID))  #n=293
dfTemp1 <- dfTemp %>%
  group_by(IP_PATIENT_ID) %>%
  summarise(max = max(RESULT, na.rm=TRUE))
length(unique(dfTemp$IP_PATIENT_ID))  #n=290
dfTble2 <- left_join(dfTble2,dfTemp1,by='IP_PATIENT_ID')
dfTble2 <- dfTble2  %>% rename("TROPONIN" = "max")
ungroup(dfTemp)
write.csv(dfTble2,"Table2v3.csv")

dfTemp <- dfLab2day[dfLab2day$COMPONENT_NAME == 'BASE EXCESS',]  #NOTE MIN VALUE
length(unique(dfTemp$IP_PATIENT_ID))  #n=290
length(unique(dfLab2day$IP_PATIENT_ID))  #n=293
dfTemp1 <- dfTemp %>%
  group_by(IP_PATIENT_ID) %>%
```

```
  summarise(max = min(RESULT, na.rm=TRUE))
length(unique(dfTemp$IP_PATIENT_ID))  #n=290
dfTble2 <- left_join(dfTble2,dfTemp1,by='IP_PATIENT_ID')
dfTble2 <- dfTble2  %>% rename("BASE EXCESS" = "max")
ungroup(dfTemp)
write.csv(dfTble2,"Table2v3.csv")

dfTemp <- dfLab2day[dfLab2day$COMPONENT_NAME == 'BICARBONATE',]  #NOTE MIN VALUE
length(unique(dfTemp$IP_PATIENT_ID))  #n=290
length(unique(dfLab2day$IP_PATIENT_ID))  #n=293
dfTemp1 <- dfTemp %>%
  group_by(IP_PATIENT_ID) %>%
  summarise(max = min(RESULT, na.rm=TRUE))
length(unique(dfTemp$IP_PATIENT_ID))  #n=290
dfTble2 <- left_join(dfTble2,dfTemp1,by='IP_PATIENT_ID')
dfTble2 <- dfTble2  %>% rename("BICARBONATE" = "max")
ungroup(dfTemp)
write.csv(dfTble2,"Table2v3.csv")

#################################################################################

################  CREAT TABLE1

 library(readxl)
Table2v3 <- read_excel("C:/Users/ehlsu/OneDrive/Research/Pneumatosis
intestinalis/Table2v3.xlsx")
#View(Table2v3)

###############  RECODE RACE/ETHNICITY

Table2v3$RACE <- sub("Do not identify with Race","Other",Table2v3$RACE)  #Combine
categoories
Table2v3$RACE <- sub("Multiple Races","Other",Table2v3$RACE)
Table2v3$RACE <- sub("Patient Refused","Other",Table2v3$RACE)
Table2v3$RACE <- sub("Unknown","Other",Table2v3$RACE)


Table2v3$ETHNICITY <- sub("Choose Not to Answer","0",Table2v3$ETHNICITY)
Table2v3$ETHNICITY <- sub("Mexican, Mexican American,
Chicano/a","1",Table2v3$ETHNICITY)
Table2v3$ETHNICITY <- sub("Hispanic/Spanish origin Other","1",Table2v3$ETHNICITY)
Table2v3$ETHNICITY <- sub("Not Hispanic or Latino","0",Table2v3$ETHNICITY)
Table2v3$ETHNICITY <- sub("Puerto Rican","1",Table2v3$ETHNICITY)
Table2v3$ETHNICITY <- sub("Puerto Rican","1",Table2v3$ETHNICITY)
Table2v3$ETHNICITY <- sub("Unknown","0",Table2v3$ETHNICITY)
Table2v3$ETHNICITY <- sub("Hispanic or Latino","1",Table2v3$ETHNICITY)


# table(Table2v3$RACE) #to get count of the various racial categories
#table(Table2v3$ETHNICITY)
#colnames(Table2v3)[5]="HISPANIC"
#Table2v3$HISPANIC <- as.numeric(Table2v3$HISPANIC)   #Convert to number type from
character



##### CREATE TABLE 1

Table2v3$AllSurg <- Table2v3$LapOnly + Table2v3$AllResect

Table2v3$Dead90 <- factor(Table2v3$Dead90, levels=c(0,1),
                          labels=c("Alive",
                                   "Dead"))
```

```
Table2v3$AGE <- as.numeric(Table2v3$AGE)
units(Table2v3$AGE) <- "Years"

Table2v3$SEX <- factor(Table2v3$SEX)
Table2v3$RACE <- factor(Table2v3$RACE)

Table2v3$ETHNICITY <- factor(Table2v3$ETHNICITY , levels=c(0,1),
                          labels=c("Not Hispanic",
                                   "Hispanic"))

Table2v3$TROPONIN <- factor(Table2v3$TROPONIN)

Table2v3$AllSurg <- factor(Table2v3$AllSurg, levels=c(0,1),
                          labels=c("No Surgery",
                                   "Surgery"))
Table2v3$LapOnly <- factor(Table2v3$LapOnly, levels=c(0,1),
                           labels=c("No Laparotomy",
                                    "EXPLORATORY LAPAROTOMY ONLY"))
Table2v3$AllResect <- factor(Table2v3$AllResect, levels=c(0,1),
                           labels=c("No Resect",
                                    "BOWEL RESECTION"))
Table2v3$WBC <- as.numeric(Table2v3$WBC)
units(Table2v3$WBC) <- "CELLS/uL"
Table2v3$LACTATE <- as.numeric(Table2v3$LACTATE)
units(Table2v3$LACTATE) <- "mg/dL"

Table2v3$CK <- as.numeric(Table2v3$CK)
units(Table2v3$CK) <- "U/L"

Table2v3$`BASE EXCESS` <- as.numeric(Table2v3$`BASE EXCESS`)
units(Table2v3$`BASE EXCESS`) <- "mmol/L"
Table2v3$BICARBONATE <- as.numeric(Table2v3$BICARBONATE)
units(Table2v3$BICARBONATE) <- "mmol/L"

Table1<-table1(~ AGE + SEX + RACE + ETHNICITY +
                 AllSurg + LapOnly + AllResect + WBC + LACTATE +
                 CK + TROPONIN  + `BASE EXCESS`  + BICARBONATE | Dead90,
               data=Table2v3,
               overall = FALSE,
               render.continuous = my.render.cont,
               extra.col=list(`SMD`=SMD_value))

Table1
#####################################################################################
#################



############## MATCH PNEUM TO NO PNEUM FOR SURVIVAL Analysis

pacman::p_load(tidyverse,tidytext,data.table,stringi,dplyr)
#setwd("C:\\Users\\ehlsu\\OneDrive\\Research\\Pneumatosis intestinalis")
setwd("C:\\Users\\ehlsu\\OneDrive\\Research\\Pneumatosis intestinalis\\Old Files")
df <- read.csv("AllAbdCT23541.csv")
df1 <- read.csv("RadFirstDate.csv")
df1 <- subset(df1,select=-X)
df2 <- left_join(df,df1,by="IP_PATIENT_ID") #add Pneum date to file
write.csv(df2,"AllAbdCT23541Pneum.csv")

df2$DEAD <- ifelse(df2$VITAL_STATUS=="Known Deceased",1,0) #Dummy variable for death
df2$PNEUM <- ifelse(is.na(df2$PNEUM_TIME),0,1)
write.csv(df2,"AllAbdCT23541Pneum.csv")  #update file with dummy variables
```

```
#df2 <- read.csv("AllAbdCT23541Pneum.csv")

df2$RESULT_TIME <- as.Date(df2$RESULT_TIME,format = "%m/%d/%Y")
df2$DEATH_DATETIME <- as.Date(df2$DEATH_DATETIME,format = "%m/%d/%Y")
length(unique(df2$IP_PATIENT_ID))   # n=7423
sum(df2$PNEUM)  # n=1863 PNEUM patients
df3 <- df2[df2$PNEUM != 1,] #df3 is nonPneum patients

######################

df2 <- df2  %>%  group_by(IP_PATIENT_ID) %>%
  filter(PNEUM==1 & RESULT_TIME==PNEUM_TIME)
ungroup(df2)  ### Remove duplicate CTs for PNEUM patients

df2 <- rbind(df3,df2) #Add back the PNEUM patients

length(unique(df2$IP_PATIENT_ID))   # n=7391
sum(df2$PNEUM)  # n=290 PNEUM patients

df2$DAYS_RAD_DEATH = df2$DEATH_DATETIME - df2$RESULT_TIME
df2 <- subset(df2,is.na(df2$DAYS_RAD_DEATH) | df2$DAYS_RAD_DEATH > -31) #Take out
implausible dates #Take out 3 cases where dates do not make sense

write.csv(df2,"AllAbdCT23541Pneum.csv")  #Repeat CTs for PNEUM patients taken out -
file has deaths but no death date that were filtered out

#####################################################################################

### ADD ELIX VRIABLES TO MATCH WORKING FILE AllAbdCT23541Pneum.csv
setwd("C:\\Users\\ehlsu\\OneDrive\\Research\\Pneumatosis intestinalis\\Old Files")
df <- read.csv("AllAbdCT23541Pneum.csv")
dfElix <- read.csv("AllCohortElix.csv")
df2 <- left_join(df,dfElix,by="IP_ENC_ID")
write.csv(df2,"AllAbdCT23541PneumElix.csv")

##################################################################################
########################### DO MATCH


pacman::p_load(tidyverse,tidytext,data.table,stringi,dplyr,MatchIt)
#setwd("C:\\Users\\ehlsu\\OneDrive\\Research\\Pneumatosis intestinalis")
setwd("C:\\Users\\ehlsu\\OneDrive\\Research\\Pneumatosis intestinalis\\Old Files")
#df <- read.csv("AllAbdCT23541PneumElix.csv")
df1 <- read.csv("AllAbdCT23541PneumElix.csv")
df <- df1[!is.na(df1$chf),]  # Take out NA rows from Elix variables

######## PRE MATCH CHECK BALANCE

library(MatchIt)
library("marginaleffects")

df$RESULT_TIME <- as.Date(df$RESULT_TIME)
df$ResTime <- as.numeric(df$RESULT_TIME)   # HAVE MULT IMAGES FOR SOME PATIENTS -
MATCH ON RESULT TIME WHICH WILL ONLY KEEP
                                            # CONTROL PATIENTS THAT HAD A CT AROUND THE
SATE OF A PNEUMATOSIS DX FOR EXP PATIENTS
m.out0 <- matchit(PNEUM ~ AGE + SEX + chf + ld + alcohol + wloss + coag + ResTime,
data = df,
                  method = NULL, distance = "glm")
summary(m.out0)


m.out1 <- matchit(PNEUM ~ AGE + SEX + ResTime, data = df,
```

```
                  method = "nearest", distance = "glm")    # Nearest Neighbor Match
m.out1
summary(m.out1, un = FALSE)

plot(m.out1, type = "jitter", interactive = FALSE)
plot(m.out1, type = "density", interactive = FALSE,
     which.xs = ~AGE + SEX + ResTime)

rio::export(summary(m.out0)[["sum.all"]], "mout.xlsx")  #Output of some parameters to
excel
rio::export(summary(m.out0)[["nn"]], "mout2.xlsx")


# https://cran.r-project.org/web/packages/MatchIt/vignettes/MatchIt.html

################### COARSENED EXACT MATCHING

m.out1 <- matchit(PNEUM ~ AGE + SEX + chf + ld + alcohol + wloss + coag + ResTime,
data = df,
        method = "cem",
        estimand = "ATT",
        s.weights = NULL,
        verbose = FALSE,
        )
m.out1
summary(m.out1)

######### FULL MODEL USED IN MS

m.out1 <- matchit(PNEUM ~ AGE + SEX + chf + carit + valv + pcd + pvd + hypunc +
                    hypc + para + ond + cpd + diabunc + diabc +
                    hypothy + rf + ld + pud +  aids + lymph +metacanc + solidtum +
                    rheumd + coag + obes + wloss + blane + dane +
                    alcohol + drug + psycho + depre + ResTime, data = df,
                  method = "full",
                  estimand = "ATT",
                  s.weights = NULL,
                  verbose = FALSE,
                  k2k = FALSE,
)                                    # EXCLUDE ELIX VARIABLE fed-fluids and
electrolyte disorder
summary(m.out1)
plot(m.out1, type = "density", interactive = FALSE,
     which.xs = ~AGE + SEX + ResTime)


### https://kosukeimai.github.io/MatchIt/reference/method_cem.html
### https://kosukeimai.github.io/MatchIt/reference/summary.matchit.html
### https://kosukeimai.github.io/MatchIt/index.html
#######################  NULL MODEL

m.out1 <- matchit(PNEUM ~ ResTime, data = df,
        method = "cem",
        estimand = "ATT",
        s.weights = NULL,
        verbose = FALSE,
        )
m.out1
summary(m.out1)

m.data <- match.data(m.out1)
head(m.data)
```

```
plot(m.out1, type = "density", interactive = FALSE,
     which.xs = ~ ResTime)

##################### Add Malahanobis

m.out2 <- matchit(PNEUM ~ AGE + SEX + ResTime, data = df,
                  method = "cem",
                  k2k = TRUE, k2k.method = "mahalanobis")

m.out2
summary(m.out2, un = FALSE)

plot(m.out2, type = "density", interactive = FALSE,
     which.xs = ~AGE + SEX + chf + ld + alcohol + wloss + coag + ResTime)

# https://kosukeimai.github.io/MatchIt/reference/method_cem.html

################# MODEL FITS  Logistic  USED FOR MS

m.data <- match.data(m.out1)
head(m.data)


fit2 <- glm(DEAD ~ PNEUM * (AGE + SEX + chf + carit + valv + pcd + pvd + hypunc +
hypc + para + ond + cpd + diabunc + diabc +
hypothy + rf + ld + pud +  aids + lymph +metacanc + solidtum +
rheumd + coag + obes + wloss + blane + dane +
alcohol + drug + psycho + depre),
data = m.data, weights = weights,
family = quasibinomial())   #exlude fluids (fed) and score from Elix


library(gtsummary)
tbl_logreg <-
  tbl_regression(fit2, exponentiate = TRUE)

tbl_logreg  # To display log regression table

###############################################
avg_comparisons(fit2,
                variables = "PNEUM",
                vcov = ~subclass,
                newdata = subset(m.data, PNEUM == 1),
                wts = "weights",
                comparison = "lnratioavg",
                transform = "exp")
##########

### MODEL FIT SURVIVAL

library(survival)

Surv <- coxph(Surv(DAYS_RAD_DEATH) ~ PNEUM, data = m.data, robust = TRUE,
              weights = weights, cluster = subclass)

summary(Surv)

sfit <- survfit(Surv(DAYS_RAD_DEATH, DEAD)~PNEUM, data=df)
plot(sfit,lty = 2:3,xlab="Days", ylab="Survival")   #Survival curve for raw data
legend(2000, .95, c("No Pneumatosis", "Pneumatosis"), lty = 2:3)


sfit <- survfit(Surv(DAYS_RAD_DEATH, DEAD)~PNEUM, weights=weights, data=m.data)
```

```
plot(sfit,lty = 2:3,xlab="Days", ylab="Survival")   #Survival curve for matched data
legend(2000, .95, c("No Pneumatosis", "Pneumatosis"), lty = 2:3)

# https://cran.r-project.org/web/packages/MatchIt/vignettes/estimating-effects.html
# coxph interpretation https://stats.stackexchange.com/questions/83892/understanding-
coxph-output-in-r
# explain LR v Wald v Score test for model testing
https://stats.oarc.ucla.edu/other/mult-pkg/faq/general/faqhow-are-the-likelihood-
ratio-wald-and-lagrange-multiplier-score-tests-different-andor-similar/

###############################################################################

### ADD ELIX TO ENCOUNTER_DIAGNOSES


library(comorbidity)


setwd('F:\\Inbound\\CTSI\\ELivingston_23_23-001036\\Analysis')
setwd('F:\\Inbound\\CTSI\\ELivingston_23_23-001036\\Data')
setwd("C:\\Users\\ehlsu\\OneDrive\\Research\\Pneumatosis intestinalis")

pacman::p_load(tidyverse,tidytext,data.table,stringi,touch,comorbidity)
df <- read.csv("Encounter_Diagnoses.csv")  #Read in diagnosis file for patients who
died
x <- subset(df,select=c(IP_ENC_ID,ICD_TYPE,ICD_CODE))
x$Convert <- ifelse(df$ICD_TYPE=="9",icd_map(df$ICD_CODE,from = 9, to =
10),df$ICD_CODE <-df$ICD_CODE)
dfe <- comorbidity(x=x, id="IP_ENC_ID", code="Convert",map = "elixhauser_icd10_quan",
assign0 = FALSE)
dfe$score <- score(x = dfe, weights = "vw", assign0 = FALSE)
write.csv(dfe,"AllCohortElix.csv")

#############################################################################
####################### CREATE TIME TO SURGERY File

pacman::p_load(tidyverse,tidytext,data.table,stringi,dplyr)
setwd("C:\\Users\\ehlsu\\OneDrive\\Research\\Pneumatosis intestinalis")
df <- read.csv("RadFirstDate.csv")
df1 <- read.csv("MasterPathProc1.csv")
df1$AnySurg <- df1$RESECT + df1$ANAST + df1$OSTOMY + df1$ADHESIONS + df1$EXLAP

length(unique(df1$IP_PATIENT_ID))
sum(df1$AnySurg)  # n=290 PNEUM patient

df1["AnySurg"][is.na(df1["AnySurg"])] <- 0  #rep

df2 <- df1 %>% group_by(IP_PATIENT_ID) %>%
   slice(which.max(AnySurg))
ungroup(df1)

df3<- subset(df2,select = c("IP_PATIENT_ID","AnySurg","Days_Rad_Proc",
                            "RESECT","ANAST","OSTOMY","ADHESIONS","EXLAP",
                            "Dead90","DeathDays"))
df4 <- left_join(df,df3,by="IP_PATIENT_ID")
write.csv(df4,"TimeToSurg.csv")


################################################################################

### DATA FOR TABLE OF VARIOUS OPERATIONS v ALIVE/DEAD
```

```
pacman::p_load(tidyverse,tidytext,data.table,stringi,dplyr)
setwd("C:\\Users\\ehlsu\\OneDrive\\Research\\Pneumatosis intestinalis")
df <- readxl::read_xlsx("TimeToSurg.xlsx",sheet='Analyze')

df48 <- df[(df$Days_Rad_Proc <=2 | is.na(df$Days_Rad_Proc)),]
#df48 <- df[(df$Days_Rad_Proc > 2) & !is.na(df$Days_Rad_Proc),]

df48$dead90 <- ifelse(df48$DeathDays <=90,'Dead90','NotDead')
df48$dead30 <- ifelse(df48$DeathDays <=30,'Dead30','NotDead')

df48  <- df48 %>%
  mutate(dead90 = coalesce(dead90, "NotDead"))  ### Replace NA with test string
df48  <- df48 %>%
  mutate(dead30 = coalesce(dead30, "NotDead"))


df48$Alive <- ifelse((df48$dead90 !='Dead90' & df48$dead30 !='Dead30'),"Alive","Dead")
df48$AnySurg <- ifelse(df48$AnySurg==1,'HadSurg','NoSurg')

table(df48$AnySurg,df48$dead90,useNA = "ifany")
table(df48$AnySurg,df48$dead30,useNA = "ifany")
table(df48$AnySurg,df48$Alive,useNA = "ifany")

df48$LapOnly <- ifelse(df48$AnyLapNoResect ==1,'HadLap','NoSurg')

table(df48$LapOnly,df48$dead90,useNA = "ifany")
table(df48$LapOnly,df48$dead30,useNA = "ifany")
table(df48$LapOnly,df48$Alive,useNA = "ifany")

df48$AnyResect <- ifelse(df48$AnyResect ==1,'HadResect','NoSurg')

table(df48$AnyResect,df48$dead90,useNA = "ifany")
table(df48$AnyResect,df48$dead30,useNA = "ifany")
table(df48$AnyResect,df48$Alive,useNA = "ifany")

table(df$Dead90,useNA = "ifany")
#####################################################################################
###### GET AUTOPSY RESULTS

pacman::p_load(tidyverse,tidytext,data.table,stringi)


setwd("C:\\Users\\ehlsu\\OneDrive\\Research\\Pneumatosis intestinalis")
setwd('F:\\Inbound\\CTSI\\ELivingston_23_23-001036\\Data')
setwd('F:\\Inbound\\CTSI\\ELivingston_23_23-001036\\Analysis')
df <- read.csv("Pathology.csv")     #7,431,222 lines for information on 16,728
patients in ULEAD
length(unique(df$IP_PATIENT_ID))  #n=16,728 patients
length(unique(df$IP_ORDER_PROC_ID))  #n=411,459 exams



df$NARRATIVE <- tolower(df$PROCEDURE_DESCRIPTION)   # make all lower case
df1 <- df %>% filter(grepl('autopsy',df$NARRATIVE))  #n=402 lines of text with the
words of interest

dfPath <- read.csv("Pathology_Results.csv")
setwd('F:\\Inbound\\CTSI\\ELivingston_23_23-001036\\Analysis')
dfTTS <- read.csv('TimeToSurg.csv')
dfTTS <- dfTTS[dfTTS$AnySurg==0,]
dfID <- subset(dfTTS,select=c('IP_PATIENT_ID','PNEUM_TIME'))
dfIDAutopsy <- inner_join(dfID,df1,by='IP_PATIENT_ID')
length(unique(dfIDAutopsy$IP_PATIENT_ID))  #n=6 patients
```

```
dfn6Pts <- subset(dfIDAutopsy,select=c('IP_PATIENT_ID','RESULT_TIME'))
dfn6Pts <- distinct(dfn6Pts,RESULT_TIME,.keep_all=TRUE)
dfn6Pts <- dfn6Pts[dfn6Pts$RESULT_TIME != "",]  #get rid of rows with blank cells
dfn6Path <- inner_join(dfn6Pts,dfPath,by='IP_PATIENT_ID')
dfPneumTime <- subset(dfTTS,select=c('IP_PATIENT_ID',"PNEUM_TIME"))
dfn6Path <- inner_join(dfn6Path,dfPneumTime,by='IP_PATIENT_ID')
dfn6Path$DAYS_PNEUM_PATH <- as.Date(dfn6Path$RESULT_TIME,format = "%m/%d/%Y") -
as.Date(dfn6Path$PNEUM_TIME)
write.csv(dfn6Path,'Autopsyn6.csv')

################################################################################
################################################################################

######### RE-RUN MATCHED SURVIVAL Analysis

pacman::p_load(tidyverse,tidytext,data.table,stringi,dplyr,MatchIt)
setwd("C:\\Users\\ehlsu\\OneDrive\\Research\\Pneumatosis intestinalis")
setwd("C:\\Users\\ehlsu\\OneDrive\\Research\\Pneumatosis intestinalis\\Old Files")
#df <- read.csv("AllAbdCT23541PneumElix.csv")
df1 <- read.csv("AllAbdCT23541PneumElix.csv")
df <- df1[!is.na(df1$chf),]  # Take out NA rows from Elix variables

library(MatchIt)
library("marginaleffects")

df$RESULT_TIME <- as.Date(df$RESULT_TIME)
df$ResTime <- as.numeric(df$RESULT_TIME)


m.out1 <- matchit(PNEUM ~ AGE + SEX + chf + carit + valv + pcd + pvd + hypunc +
                    hypc + para + ond + cpd + diabunc + diabc +
                    hypothy + rf + ld + pud +  aids + lymph +metacanc + solidtum +
                    rheumd + coag + obes + wloss + blane + dane +
                    alcohol + drug + psycho + depre + ResTime, data = df,
                  method = "full",
                  estimand = "ATT",
                  s.weights = NULL,
                  verbose = FALSE,
                  k2k = FALSE,
)                                     # EXCLUDE ELIX VARIABLE fed-fluids and
electrolyte disorder
summary(m.out1)

############ FIX PLOT ERRORS

par("mar")
par(mar=c(1,1,1,1))

### iF ERROR EXPAND THE PLOT WINDOW- SEE ARTICLE BELOW

### https://stackoverflow.com/questions/23050928/error-in-plot-new-figure-margins-too-
large-scatter-plot

###################################
plot(m.out1, type = "density", interactive = FALSE,
     which.xs = ~AGE + SEX + ResTime)

m.data <- match.data(m.out1)
head(m.data)

plot(m.out1, type = "density", interactive = FALSE,
     which.xs = ~ ResTime)
```

```
### MODEL FIT SURVIVAL

library(survival)

Surv <- coxph(Surv(DAYS_RAD_DEATH) ~ PNEUM, data = m.data, robust = TRUE,
              weights = weights, cluster = subclass)

summary(Surv)


SurvUnMatch <- coxph(Surv(DAYS_RAD_DEATH) ~ PNEUM, data = df, robust = TRUE)
# SurvUnMatch <- coxph(Surv(DAYS_RAD_DEATH,DEAD) ~ PNEUM, data = df, robust = TRUE)
#ADDING STAUS VAIABLE DEAD DOES NOT CHANGE ANYTHING

summary(SurvUnMatch)

sfit <- survfit(Surv(DAYS_RAD_DEATH, DEAD)~PNEUM, data=df)
plot(sfit,lty = 2:3,xlab="Days", ylab="Survival")   #Survival curve for raw data
legend(2000, .95, c("No Pneumatosis", "Pneumatosis"), lty = 2:3)


sfit <- survfit(Surv(DAYS_RAD_DEATH, DEAD)~PNEUM, weights=weights, data=m.data)
plot(sfit,lty = 2:3,xlab="Days", ylab="Survival")   #Survival curve for matched data
legend(2000, .95, c("No Pneumatosis", "Pneumatosis"), lty = 2:3)

#####################################################################################
########################
#####################################################################################
########################

########  Revise regression 11/13/23 Fix ResTime problem

pacman::p_load(tidyverse,tidytext,data.table,stringi,dplyr,MatchIt)

setwd("C:\\Users\\ehlsu\\OneDrive\\Research\\Pneumatosis intestinalis\\Old Files")
#df <- read.csv("AllAbdCT23541PneumElix.csv")
df1 <- read.csv("AllAbdCT23541PneumElix.csv")
df <- df1[!is.na(df1$chf),]  # Take out NA rows from Elix variables

library("marginaleffects")

df$RESULT_TIME <- as.Date(df$RESULT_TIME)
df$ResTime <- as.numeric(df$RESULT_TIME)

m.out1 <- matchit(PNEUM ~ AGE + SEX + chf + carit + valv + pcd + pvd + hypunc +
                    hypc + para + ond + cpd + diabunc + diabc +
                    hypothy + rf + ld + pud +  aids + lymph +metacanc + solidtum +
                    rheumd + coag + obes + wloss + blane + dane +
                    alcohol + drug + psycho + depre + ResTime, data = df,
                  method = "full",
                  estimand = "ATT",
                  s.weights = NULL,
                  verbose = FALSE,
                  k2k = FALSE,
)                                    # EXCLUDE ELIX VARIABLE fed-fluids and
electrolyte disorder
summary(m.out1)


m.data <- match.data(m.out1)
 head(m.data)

fit2 <- glm(DEAD ~ PNEUM * (AGE + SEX + chf + carit + valv + pcd + pvd + hypunc +
```

```
          hypc + para + ond + cpd + diabunc + diabc +
          hypothy + rf + ld + pud +  aids + lymph +metacanc + solidtum +
          rheumd + coag + obes + wloss + blane + dane +
          alcohol + drug + psycho + depre),
             data = m.data, weights = weights,
             family = quasibinomial())   #exlude fluids (fed) and score from Elix
                                        # Remove ResTime for logistic regression of
all matched data

x <- avg_comparisons(fit2,
                     variables = "PNEUM",
                     vcov = ~subclass,
                     newdata = subset(m.data, PNEUM == 1),
                     wts = "weights",
                     comparison = "lnratioavg",
                     transform = "exp")
summary(x)

library(gtsummary)
      tbl_logreg <-
         tbl_regression(fit2, exponentiate = TRUE)

         tbl_logreg

######################################################################################
#######################
```
